# Combustion Kinetics and Reaction Mechanisms of Rice Straw During Oxy-Fuel Combustion

**DOI:** 10.3390/ma19071321

**Published:** 2026-03-26

**Authors:** Dandan Li, Qing Wang, Yufeng Pei, Xiuyan Zhang, Chang Yu, Hongpeng Zhao, Da Cui, Yan Pan, Yuqi Wang

**Affiliations:** 1 Engineering Research Centre of Oil Shale Comprehensive Utilization, Ministry of Education, Northeast Electric Power University, Jilin 132012, China; lidandan@nepdi.net (D.L.); rlx888@126.com (Q.W.); yuchang@nepdi.net (C.Y.); jlcuida@163.com (D.C.); jlpanyan2025@163.com (Y.P.); 2 Northeast Electric Power Design Institute Co., Ltd., China Power Engineering Consulting Group, Changchun 130021, China; peiyufeng@nepdi.net (Y.P.); zhangxiuyan@nepdi.net (X.Z.); zhaohongpeng@nepdi.net (H.Z.); 3 School of Science, Northeast Electric Power University, Jilin 132012, China

**Keywords:** oxy-fuel combustion, physicochemical property, combustion characteristic, release of pollutants, reaction kinetics

## Abstract

Oxy-fuel combustion is a near-zero emission technology that utilizes high-concentration O_2_ in place of air, combined with recycled flue gas, to achieve efficient combustion and enable effective CO_2_ capture. In this study, air (21% O_2_/79% N_2_) was used as the control atmosphere, and rice straw combustion experiments were conducted using thermogravimetric analysis and differential scanning calorimetry and differential scanning calorimetry coupled with mass spectrometry (TG-MS) at heating rates of 10, 20, and 30 °C/min under oxy-fuel conditions of 30% O_2_/70% CO_2_, 50% O_2_/50% CO_2_, and 70% O_2_/30%CO_2_. The combustion behavior, pollutant emissions, reaction kinetics, and underlying mechanisms were systematically evaluated. The results show that CO_2_ in oxy-fuel atmospheres exhibits a higher thermal inertia, due to its greater density and specific heat capacity, thereby enhancing flame stability. Oxy-fuel atmospheres reduce the ignition temperature (*Tᵢ*) and burnout temperature (*T_f_*), shorten the combustion duration, shift DTG and DSC peaks to lower temperatures, and result in sharper peaks along with an increased ignition index (*Cᵢ*), burnout index (*C_b_*), and comprehensive combustion index (*S*). Mass spectrometry (MS) analysis reveals that oxy-fuel atmospheres combined with heating rates of 20–30 °C/min suppress O_2_ diffusion and thermal NO formation, reducing NO_x_ emissions by over 75% and simultaneously inhibiting the release of SO_2_ and COS. Kinetic analysis using the FWO and Friedman methods shows that the activation energy decreases from 210.5 kJ/mol and 219.1 kJ/mol under air conditions to 110.5 kJ/mol and 114.6 kJ/mol in oxy-fuel atmospheres, representing a reduction in reaction barriers of 47.5% and 47.7%, respectively. The reaction mechanisms were identified as three-dimensional diffusion-controlled processes at heating rates of 20–30 °C/min, and random nucleation followed by growth under high O_2_ concentration conditions at a heating rate of 30 °C/min. Optimizing the combustion atmosphere and heating rate enhances the rice straw combustion efficiency and reduces pollutant emissions, thereby providing theoretical support for its clean and efficient utilization.

## 1. Introduction

Oxy-fuel combustion represents a significant research focus in the clean energy field, building upon the theoretical foundations established by Abraham in 1982 [1]. Moreover, the core technology suggests that the transformation of the conventional “Air-N_2_” atmosphere into an “O_2_-recycled flue gas (primarily consisting of CO_2_)” The environment induces fundamental alterations at the levels of thermodynamics, heat and mass transfer, and reaction kinetics, while significantly increasing the oxygen concentration in the combustion atmosphere; the efficient enrichment and recycling of CO_2_ in the flue gas were achieved. Furthermore, by integrating denitration with flue gas recirculation, the proportion of CO_2_ in the flue gas can be elevated to nearly 90% [2], thereby significantly simplifying the subsequent carbon capture and storage process. In light of the flue gas recirculation capabilities, recirculated flue gas regulates the furnace temperature, enhances the combustion efficiency, reduces flue gas losses and pollutant emissions, and achieves high-performance, environmentally sustainable combustion. The transition to a low-carbon energy landscape necessitates more than carbon capture; it requires a profound understanding of the pollutant chemistry under non-conventional atmospheres and a multi-dimensional validation of techno-economic feasibility [3,4]. By addressing these critical scientific and economic bottlenecks, these technologies offer a commercially viable trajectory for the clean utilization of conventional fuels, thereby providing high-impact engineering value for the decarbonization of energy-intensive industries.

The fundamental physicochemical properties of the reaction atmosphere play a decisive role in combustion performance. The O_2_ content in ambient air is approximately 21%, while N_2_ accounts for about 79% [5]. The oxy-fuel combustion technology uses a high concentration of oxygen instead of air as the oxidizer, and introduces the captured carbon dioxide from power plants to replace the nitrogen used in traditional combustion as the main diluent, thereby creating an O_2_/CO_2_ combustion environment with a high oxygen concentration. Understanding the distinctive dependence of thermodynamic and transport properties on the CO_2_ or N_2_ composition is critical, as these molecular traits dictate the pore-scale occupancy and interphase exchange rates that are often overlooked in conventional models [6,7,8,9]. Such molecular-level sensitivity fundamentally steers the momentum and energy diffusion during complex combustion or gasification, posing a significant challenge for the precise control and optimization of high-efficiency energy systems. Therefore, the variation characteristics of thermophysical parameters of different atmospheres are of crucial significance for revealing their influence mechanisms on combustion efficiency, flame stability, and reaction kinetics [10]. In recent years, the significant biomass oxy-fuel combustion technology indicates considerable attention owing to the potential for achieving low-carbon emissions and efficient energy utilization. Shan et al. [11] demonstrated that, in oxy-fuel combustion experiments involving Pinus bungeana and rice husk, when the O_2_ concentration exceeded 21%, ignition was advanced, the internal ignition temperature decreased, and the volatile combustion duration shortened with increasing O_2_ levels; however, when the O_2_ concentration surpassed 50%, the rate of reduction in combustion time gradually diminished, indicating a saturation effect. Dai et al. [12,13] reported that, in oxy-fuel biomass combustion, NOx emissions decreased with increasing O_2_ concentration, and NO emissions alone were reduced by approximately 48%. Meng et al. [14] demonstrated that, in a simulated oxy-fuel combustion environment, replacing the baseline atmosphere of 21% O_2_/79% N_2_ with a 30% O_2_/70% CO_2_ mixture significantly enhanced the combustion efficiency for dry distillers’ grains with solubles and rice husks, while effectively reducing emissions of N_2_ and sulfur-based pollutants. Guo et al. [15] conducted experimental investigations on the oxy-fuel combustion of corn stalks and rice husks under varying heating rates and performed a comprehensive kinetic analysis. The results revealed that, at low heating rates, the combustion reactions of corn stalk and rice husk biochar in a pure O_2_ atmosphere exhibited a higher reactivity compared to those in an air atmosphere, with activation energies decreased by 13.08 kJ/mol and 8.79 kJ/mol, and reaction orders of 1.284 and 1.325, respectively. Moreover, at high heating rates, the activation energies for the volatile release from corn stalks and rice husks were determined to be 74.76 kJ/mol and 85.66 kJ/mol, respectively. Therefore, the important kinetic analysis conducted at low heating rates provides valuable insights into the dynamic variation pattern of the activation energy during combustion, whereas an analysis performed at high heating rates more effectively captures the intrinsic characteristics of the reaction. However, for the oxy-fuel combustion process, extensive studies have been conducted by previous researchers on fundamental experiments, such as thermogravimetric analysis and differential scanning calorimetry and differential scanning calorimetry coupled with mass spectrometry under varying heating rates. Nevertheless, a notable gap in the research remains that systematically integrates the physical and chemical properties of different atmospheric gases (including thermodynamic parameters and heat and mass transfer characteristics), synthesizes experimental data through a comprehensive analysis, and subsequently develops a universally applicable reaction kinetics model with broad adaptability via optimal function screening.

The oxy-fuel combustion of biomass, as a key path to achieving negative carbon emissions, has shown distinct evolving patterns and technical emphases in different regions at home and abroad. The pioneering international research in this field, particularly during the late 20th century, was underpinned by sophisticated forest regeneration management and policy frameworks in Northern Europe and North America [16,17]. Utilizing woody biomass such as chips and pellets, these early studies transcended simple combustion; they focused on the synergy between long-term ecological sustainability and industrial-scale engineering verification, establishing a robust foundation for integrated biomass-to-energy systems. For instance, Germany’s Schwarze Pumpe large-scale demonstration project conducted a comprehensive techno-economic assessment of the full carbon capture, transport, and storage (CCUS) chain—specifically evaluating the feasibility, operational stability, and scalability of integrating large-scale circulating fluidized bed (CFB) boilers into oxy-fuel combustion systems. This effort confirmed a robust and sustained boiler performance under oxygen-rich conditions, providing critical empirical validation for the oxy-fuel combustion of biomass as a scalable negative emission technology [18]. In contrast, although domestic research began in the 21st century and was somewhat behind the international level, it has achieved rapid progress driven by the ‘dual carbon’ goal. The research direction has focused more on the mechanism exploration of complex component fuels and the development of key equipment. The high AAEM concentration in Chinese crop straw presents a unique thermochemical bottleneck in oxy-fuel combustion, where high CO_2_ concentrations drastically modify the ash melting characteristics and mineral partitioning [19,20,21]. Research suggests that the interplay between fuel-bound alkalis and the carbon-rich atmosphere induces more severe slagging and heat transfer degradation than witnessed in wood-based systems, posing a fundamental challenge for the predictive modeling of ash deposition in next-generation power plants.

In order to effectively bridge the gap between basic theories and large-scale engineering applications, Cui et al. [22] conducted systematic research covering the laboratory, pilot-scale, and industrial-scale levels for CFB boilers ranging from 0.1 MW to 600 MW. Through an in-depth analysis of the furnace structure, gas–solid dynamics, combustion heat transfer characteristics, and emission efficiency, the results indicated that precise laboratory kinetic data is of irreplaceable value in guiding the air distribution design and heat transfer surface layout of industrial boilers. This leap from mechanism to application not only is aimed at achieving an immediate reduction in pollutants such as nitrogen and sulfur, but also involves a systematic analysis of the physical and chemical characteristic changes in ash produced by oxy-fuel combustion, providing scientific support for returning the ash to the fields as high-quality fertilizer or converting it into building materials [23]. This comprehensive consideration of the resource utilization throughout the entire life cycle not only validates the environmental friendliness of this technical approach, but also provides a solution that is both practically relevant and of engineering guidance significance for the flexibility transformation of coal-fired power plants and the coupled power generation with biomass in China.

**Figure 1:**
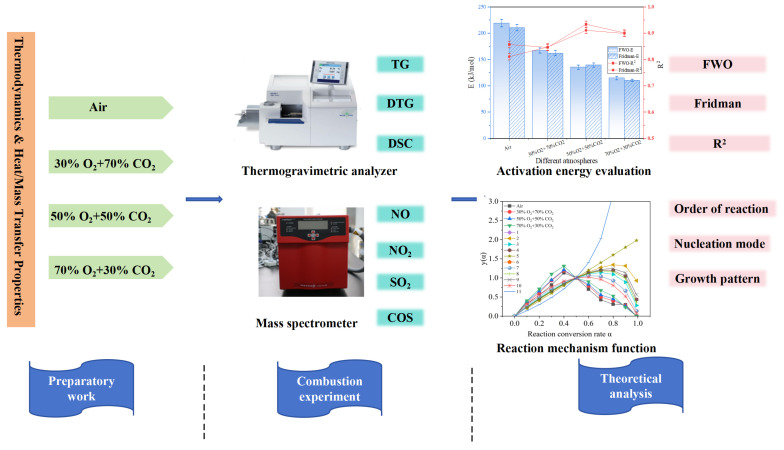
Flowchart of oxy-fuel combustion of RS under different reaction atmospheres.

This study systematically investigates the combustion characteristics of rice straw (RS) across various atmospheres including air and three oxy-fuel conditions (30% O_2_/70% CO_2_, 50% O_2_/50% CO_2_, and 70% O_2_/30% CO_2_) through an integrated experimental and theoretical framework. The specific process is shown in Figure 1. The primary scientific innovation lies in its departure from traditional kinetic studies; rather than focusing solely on chemical reaction rates, this research is the first to deeply integrate the thermophysical transport properties of the atmosphere (such as thermal diffusivity, momentum diffusion, and the Prandtl number) with multi-dimensional TG-DSC-MS data. This approach elucidates the coupling mechanism between the physical environment and chemical kinetics, specifically revealing how the high thermal inertia and low diffusivity of CO_2_ intervene in the combustion process by altering the heat and mass transfer boundary layers. Based on these theoretical foundations, thermogravimetric analysis and differential scanning calorimetry and differential scanning calorimetry and mass spectrometry were employed to monitor the combustion behavior and the release patterns of pollutants (NO_x_, SO_2_, and COS), while kinetic methods such as FWO and Friedman were applied to determine the activation energy and optimal mechanism functions. Ultimately, by bridging the laboratory-scale parameters with industrial-scale applications such as furnace selection, heat exchange surface verification, and alkali metal corrosion prevention, this research provides a solid scientific foundation for biomass oxy-fuel technology, facilitating a strategic transition from ‘carbon reduction’ to ‘negative carbon operation’ in power plants.

## 2. Materials and Methods

### 2.1. Materials and Basic Property Analysis

The agricultural residue rice straw collected from Northeast China was used as the raw material in this study. Following collection, the samples were washed with deionized water to remove surface impurities such as dust and soil. The cleaned straw was subsequently dried in a hot air oven at 105 °C for 24 h to ensure complete moisture removal. The dried material was then ground for 60 s using a mechanical grinder (Hunan Sundy Science and Technology Co., Ltd., Changsha, China) and sieved through a 140-mesh screen. The resulting fine powder that passed through the sieve was collected as the experimental sample, stored in sealed containers prior to use, and designated as RS.

The proximate analysis of samples was conducted in strict accordance with Chinese National Standards GB/T 212-2008 [24], using a SDLA718 Proximate Analyzer (Hunan Sundy Science and Technology Co., Ltd., Changsha, China). Given that ultimate analysis was performed, the contents of C, H, and N were determined accordance with Chinese National Standards GB/T 30733-2014 [25], using an Italian Euro Vector EA3000 Automatic Elemental Analyzer (EuroVector S.p.A., Milan, Lombardy, Italy). Sulfur content was analyzed using a SDS350 Infrared Sulfur Determinator (Hunan Sundy Science and Technology Co., Ltd., Changsha, China). The higher heating value (HHV) was measured using an SDC311 O_2_ Bomb Calorimeter (Hunan Sundy Science and Technology Co., Ltd., Changsha, China).

Nevertheless, to further characterize the significant biomass structural features, the samples were subjected to treatment using the Van Soest washing method with acid detergent and neutral detergent solutions. In light of sequential determination, the contents of Acid Detergent Fiber (ADF), Neutral Detergent Fiber (NDF), and related components were examined [26]. The basic physicochemical properties and proximate analysis results of the samples are summarized in Table 1, and the detergent component analysis results are listed in Table 2.

To assess the slagging risk during boiler operation, the chemical composition of RS ash was analyzed. Specifically, the ash was prepared by heating the biomass at 800 °C for 4 h in a muffle furnace and sieving the residue to below 100 mesh. Elemental scanning was then performed using an XRF analyzer (Bruker Daltonik GmbH, Bremen, Germany), with the resulting metal oxide proportions summarized in Table 3. To characterize the pore structure, nitrogen adsorption–desorption isotherms of the RS were measured. This analysis was conducted at 77 K using a TriStar II 3020 analyzer (Micromeritics Instrument Corporation, Norcross, GA, USA). These measurements provide the physical data (such as specific surface area and porosity) necessary for evaluating the heat and mass transfer characteristics of the biomass during combustion.

### 2.2. TG-DSC-MS Experiments

In this experiment, thermogravimetric analysis and differential scanning calorimetry and differential scanning calorimetry were employed to investigate the combustion behavior of RS under different oxy-fuel atmospheres. The atmosphere conditions included air and three O_2_/CO_2_ ratios (30% O_2_/70% CO_2_, 50% O_2_/50% CO_2_, and 70% O_2_/30% CO_2_), with a total atmosphere flow rate of 100 mL/min and a N_2_ protective gas flow rate of 50 mL/min. Thermogravimetric tests were conducted in the temperature range of 85–800 °C at heating rates of 10, 20, and 30 °C/min, respectively. Simultaneously, a mass spectrometer (Pfeiffer Vacuum GmbH, Asslar, Hesse, Germany) was used to monitor gas evolution. The combustion stages, weight loss characteristics, exothermic behavior, and kinetic laws under different conditions were systematically analyzed by combining TG, DTG, and DSC curves.

To comprehensively evaluate the combustion characteristics of RS, this study employs the ignition index (*C_i_*) [27,28], burnout index (*C_b_*) [29], and comprehensive combustion characteristic index (*S*) [30] as evaluation indicators. *C_i_* reflects the ignition performance at the initial stage of combustion, and *C_b_* characterizes the burnout ability at the later stage of combustion. Higher values of these two indices, respectively, indicate stronger ignition and burnout performance of the samples. *S* is used to evaluate the overall performance of the entire combustion process, and a higher value of *S* represents more complete and stable combustion. The mentioned parameters can all be calculated based on thermogravimetric (TG/DTG) data and relevant formulae [31,32]:

(1) $$C_{i}=\frac{{\left(dw/dt\right)}_{\max}}{t_{i}t_{\max}}$$

(2) $$C_{b}=\frac{{\left(dw/dt\right)}_{\max}}{\Delta t_{1/2}t_{\max}t_{b}}$$

(3) $$S=\frac{R}{E}{\left[\frac{d\left(\frac{dw}{dt}\right)}{dT}\right]}_{T=T_{i}}\cdot \frac{{\left(dw/dt\right)}_{\max}}{{\left(dw/dt\right)}_{T=T}}\cdot \frac{{\left(dw/dt\right)}_{mean}}{T_{b}}=\frac{{\left(dw/dt\right)}_{\max}{\left(dw/dt\right)}_{mean}}{T_{i}^{2}T_{b}}$$

In the calculation of combustion characteristic indices, ${\left(dw/dt\right)}_{\max}$ represents the maximum reaction rate, corresponding to the peak of the DTG curve; ${\left(dw/dt\right)}_{mean}$ is the average reaction rate between the ignition point and burnout point, reflecting the overall reaction intensity of the combustion process; $t_{i}$ is the ignition time, defined as the time elapsed from the start of the experiment until reaching the ignition temperature; $t_{\max}$ is the time corresponding to the maximum reaction rate; $t_{b}$ is the burnout time, defined as the time elapsed from the start of the experiment until reaching the burnout temperature; $\Delta t_{1/2}$ corresponds to $(dw/dt)/{\left(dw/dt\right)}_{\max}=1/2$, the time when the reaction progress reaches 50%; $T_{i}$ is the ignition temperature, typically determined as the extrapolated onset point of the TG curve or by a specific method from the DTG curve; and $T_{b}$ is the burnout temperature, generally taken as the temperature where the weight loss curve levels off. This study ensured the reliability and reproducibility of the experimental data through rigorous experimental design and high-precision instrumentation. All thermogravimetric (TGA) and differential scanning calorimetry (DSC) measurements were performed synchronously using a TGA/DSC simultaneous thermal analyzer (Mettler-Toledo AG, Greifensee, Zurich, Switzerland), featuring a balance sensitivity of 0.1 mg that enables accurate detection of minute mass changes and a temperature control accuracy of ±0.5 K. Complementary real-time evolved gas analysis was conducted using a mass spectrometer (Pfeiffer Vacuum GmbH, Asslar, Hesse, Germany) under vacuum conditions, with detection limits for most gaseous species below 100 ppb. The experiments under all conditions are independently repeated three times each to eliminate random errors. The subsequent analyses are all presented using the average value of the results from the three experiments.

### 2.3. Reaction Kinetics and Mechanism

#### 2.3.1. Kinetic Calculation Methods

The kinetic behavior of the RS combustion process was evaluated using the FWO (Flynn–Wall–Ozawa) integral method and Friedman differential method, based on non-isothermal thermogravimetric data obtained at different heating rates and under different atmospheres. RS combustion, as a typical solid heterogeneous reaction, follows the Arrhenius law, and its kinetic process can be studied using analytical methods similar to those applied to coal combustion [31].

The FWO method is an integral isoconversional method that does not require prior assumption of a reaction mechanism function. This method utilizes the linear relationship between *ln*(*β*) and 1/*T* corresponding to the same conversion rate at different heating rates, and the apparent activation energy is obtained by fitting the slope. However, due to the approximate treatment of the temperature integral term, the resulting activation energy has certain deviations [32,33,34].

The Friedman method is a differential isoconversional method. This method directly utilizes the linear relationship between *ln*(*β dα*/*dt*) and 1/*T* corresponding to the same conversion rate at different heating rates, and the apparent activation energy is obtained directly by fitting the slope [35,36]. As this method does not rely on assumptions about the reaction mechanism function, it can effectively avoid calculation deviations in activation energy caused by inappropriate selection of the mechanism function [37].

The combined use of both methods allows for cross-validation of the consistency of the calculated activation energy and provides a more comprehensive revelation of the kinetic characteristics of the RS combustion process from both integral and differential perspectives.

To calculate the kinetic parameters of the RS combustion process, the combustion reaction conversion rate is defined as α:

(4) $$\alpha =\frac{W_{0}-W}{W_{0}-W_{\infty }}$$

*W*_0_—initial mass of RS, mg;
*W_∞_*—final residual mass of RS, mg;
*W*—mass of RS at reaction time, mg.

This study systematically investigates the variation of activation energy (*E*) and determination coefficient (*R*^2^) as a function of conversion degree (*α*) under air and various O_2_/CO_2_ mixed atmospheres using the FWO and Friedman methods. To assess the reliability of the kinetic parameters, a rigorous statistical evaluation was conducted, incorporating residual error analysis, linear regression modeling, and 95% confidence and prediction intervals. All experimental data were independently acquired by the authors. While Gemini 3 served as an auxiliary tool for graphical conceptualization and figure refinement.

#### 2.3.2. Reaction Mechanism Model

In thermal analysis kinetics, the expression of the kinetic function f(α) corresponding to the RS combustion process is as follows:

(5) $$f(\alpha )={\left(1-\alpha \right)}^{n}$$

In Equation (5), α denotes the conversion rate, and n represents the reaction order. Different values of n correspond to distinct reaction mechanism models, which can be employed to describe various kinetic behaviors during combustion, including variations in the reaction interface, diffusion-controlled processes, and phase-boundary-controlled reactions.

To accurately infer the mechanism function f(α) and its integral form G(α) applicable to this process, the Malek method was employed for model discrimination [38]. This method identifies the most probable mechanism by matching the defined normalized function y(α) with standard theoretical curves. Specifically, by combining the differential form of the reaction rate Equation (6) based on the Arrhenius formula with the Coats–Redfern integral Equation (7), the expression for the integral mechanism function (8) can be derived. Subsequently, the y(α) curve is calculated by combining this with experimental data and compared with standard curves of typical mechanism functions to ultimately determine the most suitable kinetic model:

(6) $$\frac{d\alpha }{dt}=A\exp(-\frac{E}{RT})f(\alpha )$$

(7) $$\int_{0}^{\alpha }\frac{d\alpha }{dt}=G(\alpha )=\frac{ART^{2}}{E\beta }\exp(-\frac{E}{RT})$$

(8) $$G(\alpha )=\frac{RT^{2}}{E\beta }\frac{d\alpha }{dt}\frac{1}{f(\alpha )}$$

When α = 0.5, Equation (9) can be obtained:

(9) $$G(0.5)=\frac{RT_{0.5}^{2}}{E\beta }(\frac{d\alpha }{dt}{)}_{0.5}\frac{1}{f(0.5)}$$

*T*_0.5_—reaction temperature when α is 0.5;
(*dα*/*dt*)_0.5_—reaction rate when α is 0.5.

Dividing Equation (7) by (8) yields the expression for the experimental observation calculation formula y(α)_exp_ and theoretical prediction calculation formula y(α)_theo_:

(10) $$y{\left(\alpha \right)}_{\exp}=(\frac{T}{T_{0.5}})2\frac{\left(\frac{d\alpha }{dt}\right)}{{\left(\frac{d\alpha }{dt}\right)}_{0.5}}$$

(11) $$y{\left(\alpha \right)}_{\mathrm{theo}}=\frac{f(\alpha )G(\alpha )}{f(0.5)G(0.5)}$$

The matching degree between the experimental curve and the theoretical curve is calculated using the matching coefficient ${\;R}_{M}^{2}$, which is used to measure the matching degree of the nonlinear curve. The closer the value of $R_{M}^{2}$ is to 1, the more accurately the theoretical model can describe the reaction process observed in the experiment:

(12) $$R_{M}^{2}=1-\frac{\sum_{i=1}^{n}(y(\alpha {)}_{exp,i}-y(\alpha {)}_{theo,i}{)}^{2}}{\sum_{i=1}^{n}(y(\alpha {)}_{exp,i}-{\bar{y}}_{exp}{)}^{2}}$$

$y(\alpha {)}_{exp,i}$—the experimental observation value under a certain conversion rate α;
  $y(\alpha {)}_{theo,i}$—theoretical model prediction value under a certain conversion rate α;
${\bar{y}}_{exp}$—the average value of the experimental results under a certain conversion rate α.

## 3. Results and Discussion

### 3.1. Basic Physicochemical Properties

Variations in fundamental physical parameters across different combustion atmospheres substantially indicate that these critical factors influence the subsequent combustion behavior. Moreover, the conventional air demonstrates that the O_2_ concentration appears to be approximately 21%, with N_2_ constituting the remaining 79%. In contrast, oxy-fuel combustion technologies utilize recycled CO_2_ from power plant emissions. Thus, CO_2_ replaces N_2_ as the diluent gas, thereby altering the reaction environment. However, CO_2_ exhibits significant differences from N_2_ in terms of thermodynamic properties and heat transfer characteristics. Given that these properties differ substantially, oxy-fuel combustion fundamentally distinguishes from conventional combustion [10].

**Figure 2:**
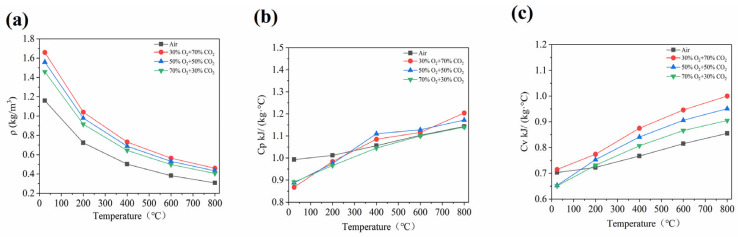
Thermodynamic properties of different reaction atmospheres at different temperatures (1 atm): (**a**) the variation pattern of *ρ*; (**b**) the variation pattern of *C_p_*; and (**c**) the variation pattern of *C_v_*.

The comparative analysis suggests that the thermodynamic properties of air and the oxy-fuel atmospheres across the range of temperatures at 1 atm are presented in Figure 2. The important thermodynamic parameters, including the density (*ρ*), the specific heat at constant pressure (*C_p_*), and the specific heat at constant volume (*C_v_*), demonstrate that these values are consistently lower for air compared to three distinct oxy-fuel atmospheres: 30% O_2_/70% CO_2_, 50% O_2_/50% CO_2_, and 70% O_2_/30% CO_2_. Given that CO_2_ molecules possess a relatively high molecular weight and a substantial molar heat capacity, this phenomenon is attributed to these molecular characteristics. Moreover, compared to N_2_, the primary component of air, CO_2_ indicates not only a higher density but also an enhanced excitation of molecular vibrational degrees of freedom at elevated temperatures, which substantially increases the overall heat capacity of the mixture [39,40]. However, a high CO_2_ content appears to lead to pronounced thermal inertia, resulting in slower temperature responses under identical heat input conditions and corresponding reduction in the overall heat transfer rate. Thus, these characteristics have significant implications for non-isothermal reaction processes involving combustion or high-temperature catalysis.

Nevertheless, in the lower temperature range, certain thermodynamic properties deviate from the general trend. Specifically, below 400 °C, the significant *C_p_* value of air exceeds that of the 30% O_2_/70% CO_2_ mixture, and, below 200 °C, its *C_v_* also surpasses the corresponding value. In light of the reduced molecular vibrational activation at low temperatures, this suggests the vibrational modes of CO_2_ molecules are not fully activated, leading to a reduced overall heat capacity. Additionally, N_2_ and O_2_, the primary components of air, exhibit relatively high heat capacities at low temperatures owing to their translational and rotational degrees of freedom. However, the thermodynamic behavior of CO_2_ appears to demonstrate a pronounced temperature dependence. Furthermore, under high-temperature conditions, the high heat capacity of CO_2_ dominates the system’s thermal behavior, which is characterized by a pronounced thermal inertia and enhanced thermal buffering capacity [41]. Although dominant at elevated temperatures, this effect diminishes significantly under low-temperature conditions, and the thermal behavior gradually converges toward that of lighter gases without being superseded by them.

In an oxy-fuel environment, the important thermodynamic properties of O_2_ and CO_2_ mixtures indicate that these parameters vary significantly with composition. As illustrated in Figure 2, an increase in O_2_ concentration accompanied by a decrease in CO_2_ leads to a reduction in key thermodynamic parameters, including density (*ρ*), isobaric specific heat (*C_p_*), and isochoric specific heat (*C_v_*). Moreover, the trend arises from the molecular weight of CO_2_ being approximately 44 g/mol, significantly higher than that of O_2_. Given that the CO_2_ proportion decreases within the gas mixture, the results show a corresponding reduction in the average molecular weight, thereby directly reducing the density (*ρ*). However, under identical temperature and pressure conditions, the density (*ρ*) of a gas appears directly proportional to its average molecular weight (*Mᵣ*). Thus, an increase in the O_2_ mole fraction leads to a reduction in the overall density of the gas mixture. Nevertheless, the significant empirical evidence demonstrates that CO_2_ exhibits a higher heat capacity, greater thermal inertia, an elevated ignition temperature, and a reduced flame temperature compared to typical oxidizing gases. Furthermore, as lower-molecular-weight O_2_ replaces higher-molecular-weight CO_2_, a species with greater vibrational contributions to the heat capacity, the reduction in the high-heat-capacity component in the gas mixture lowers both the specific heat at constant pressure (*C_p_*) and the specific heat at constant volume (*C_v_*), thereby enhancing the system’s thermal response rate. The trends of these thermodynamic parameters are in line with those described in Reference [10]. In light of these findings, the results show this facilitates rapid heating in high-temperature applications, leading to elevated combustion temperatures and accelerated reaction kinetics. In practical engineering applications such as combustion power plants, the proportion of O_2_ introduced into the system must be optimized according to specific operating conditions and performance objectives. Higher O_2_ concentrations do not necessarily yield a better performance.

**Figure 3:**
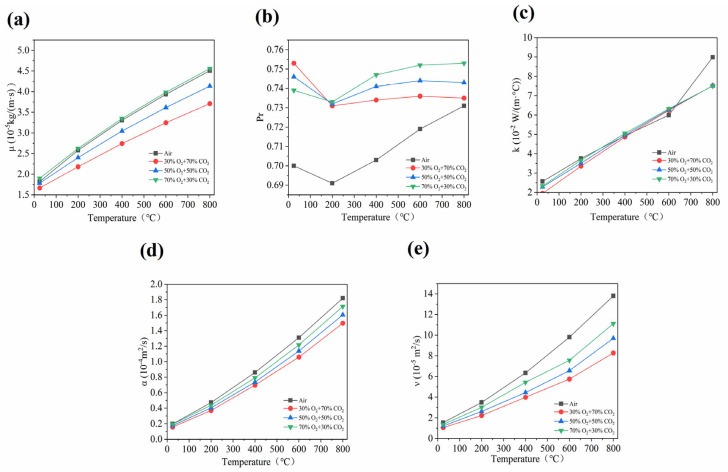
Heat and mass transfer properties of different reaction atmospheres at different temperature conditions (1 atm): (**a**) the variation pattern of *μ*; (**b**) the variation pattern of *ν*; (**c**) the variation pattern of *k*; (**d**) the variation pattern of *a*; and (**e**) the variation pattern of Pr.

A comparative analysis of the heat and mass transfer characteristics between air and oxy-fuel atmospheres under varying temperature conditions at 1 atm is presented in Figure 3. The air atmosphere exhibits higher values of dynamic viscosity (*μ*), kinematic viscosity (*ν*), thermal conductivity (*k*), and thermal diffusivity (*a*) compared to three specified oxy-fuel atmospheres, specifically 30% O_2_/70% CO_2_, 50% O_2_/50% CO_2_, and 70% O_2_/30% CO_2_. In contrast, the Prandtl number (Pr) of air is lower than that observed in the oxy-fuel atmospheres under investigation. These changes in heat and mass transfer properties are in line with those described in Reference [10]. This phenomenon can be primarily attributed to the lower molecular weight of N_2_, the main constituent of air, compared to that of CO_2_, as well as to the relatively weaker intermolecular forces in air. Consequently, the efficiency of the momentum and energy transfer is enhanced. Under identical temperature conditions, air demonstrates higher values of dynamic viscosity (*μ*) and thermal conductivity (*k*). The kinematic viscosity (*ν*) and thermal diffusivity (*a*) reasonably serve as critical parameters for characterizing the significant momentum and thermal diffusion capabilities [42,43]. Moreover, N_2_ demonstrates a lower density than CO_2_; however, its dynamic viscosity (*μ*) shows higher values, thus leading to increased kinematic viscosity (*ν = μ*/*ρ*) and thermal diffusivity (*a* = *k*/*ρC_p_*). Given that the momentum and thermal diffusion rates require characterization, the Prandtl number (*Pr* = *ν*/*a*) indicates a critical dimensionless parameter [44]. Nevertheless, both the kinematic viscosity (*ν*) and thermal diffusivity (*a*) of air show higher values than those in oxy-fuel atmospheres. However, the proportionally greater increase in thermal diffusivity leads to a reduced Pr value. This indicates that heat transfer predominates in air, whereas momentum transfer dominates in oxy-fuel atmospheres. Furthermore, thermal diffusion lags significantly behind momentum diffusion, thus resulting in the slower development of the thermal boundary layer compared to the hydrodynamic boundary layer. Additionally, this disparity substantially impairs the convective heat transfer efficiency. In light of these thermal characteristics, the temperature uniformity and mixing performance in power plant boiler furnaces appear affected. These characteristics indicate critically important factors for the engineering design and operational stability of non-isothermal flow systems and combustion processes [45].

The heat and mass transfer characteristics of gas mixtures indicate that significant variations emerge in oxy-fuel environments when the O_2_/CO_2_ concentration ratios are altered. Moreover, as the O_2_ concentration increases and the CO_2_ concentration decreases, the key parameters demonstrate shifts in the transport properties. Furthermore, Figure 3 suggests the dynamic viscosity (*μ*), kinematic viscosity (*ν*), thermal conductivity (*k*), thermal diffusivity (*a*), and Prandtl number (Pr) show an increasing trend. However, this behavior appears primarily attributed to the CO_2_ molecular weight and collision cross section. Nevertheless, CO_2_ intermolecular forces impede momentum and energy transfer efficiency [46]. Thus, the O_2_ replacement of CO_2_ diminishes the inhibitory effects on transport processes. Additionally, the molecular average velocity increases at the given temperature. Given that momentum exchange between molecules becomes more frequent, the transport capacity increases and heat transfer rates improve. In light of the enhanced transfer rates, the combustion intensity and temperature uniformity show improvement.

The fundamental physicochemical properties of air and oxy-fuel atmospheres indicate that their distinct and complementary behaviors reflect differing roles in the heat, mass, and momentum transfer processes. Air, characterized by a high transport efficiency, facilitates reactant mixing and enhances heat transfer. In contrast, an oxy-fuel atmosphere with an elevated CO_2_ content exhibits a superior heat storage capacity and temperature stability. The higher heat capacity contributes to an improved temperature uniformity and strengthens the thermal buffering effect during the combustion process. Therefore, in the practical design of combustion systems for RS, a comprehensive consideration of the interaction between thermodynamic properties and transport characteristics is essential for the systematic optimization and control of the combustion reaction process.

### 3.2. Thermogravimetric Analysis and Differential Scanning Calorimetry and Differential Scanning Calorimetry of RS

**Figure 4:**
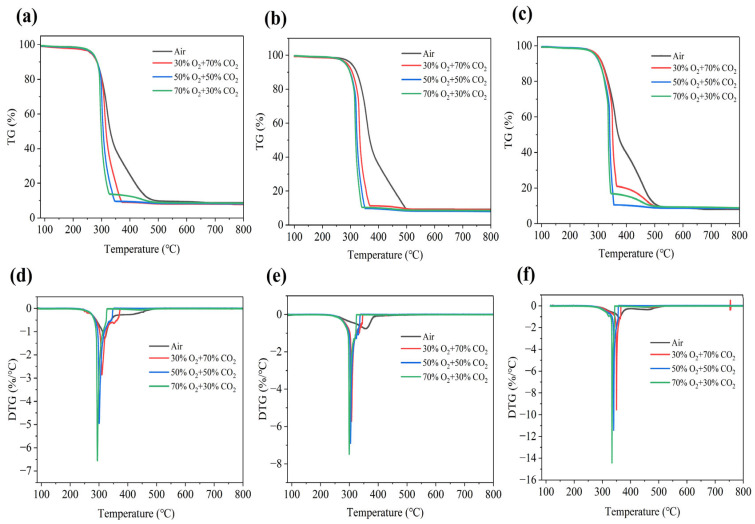
TG/DTG curves of different reaction atmospheres under different temperature conditions: (**a**–**c**) represent the TG changes at 10, 20, and 30 °C/min, respectively; and (**d**–**f**) represent the DTG changes at 10, 20, and 30 °C/min, respectively.

Thermogravimetric analysis and differential scanning calorimetry and differential scanning calorimetry (TG-DSC) is a reliable method for assessing the combustion behavior of fuels and evaluating their thermal stability through the real-time monitoring of mass changes during the combustion process [47]. Figure 4 presents the thermogravimetric (TG) and derivative thermogravimetric (DTG) curves of rice straw (RS) obtained under different heating rates (10 °C/min, 20 °C/min, and 30 °C/min) and in various gaseous atmospheres, including air, 30% O_2_/70% CO_2_, 50% O_2_/50% CO_2_, and 70% O_2_/30% CO_2_. The combustion process can be broadly divided into four distinct stages: the initial decomposition stage, the volatile matter release and ignition stage, the fixed carbon combustion stage, and the burnout stage [47]. The inorganic constituents of RS ash primarily comprise alkali and alkaline earth metals (AAEMs), including K_2_O, GaO, Na_2_O, MgO, and Al_2_O_3_, as detailed in Table 3. Below 200 °C, this process enters the initial stage of combustion. The determination of this stage is based on a mass loss rate of approximately 5%, mainly attributed to water evaporation and the volatilization and release of a small amount of AAEMs. The potassium and sodium in RS exist mostly in water-soluble ionic form. During the vigorous evaporation of water at 100–105 °C, the resulting steam flow entrains dissolved alkali metal ions from the biomass matrix, transporting them in the form of fine droplets or aerosols. The AAEMs contained in RS mainly exist in the form of carboxylates as calcium (Ca) and magnesium (Mg). At a temperature of around 150 °C, hemicellulose begins to undergo the initial pre-decomposition reaction. At this time, some AAEMs with relatively weak binding forces to carboxyl groups will migrate or redistribute along with the removal of small molecule gases such as CO_2_. Although the overall content of volatile metal compounds is extremely low, under specific conditions, metal chlorides (such as NaCl and KCl) will significantly increase their apparent vapor pressure under the action of water vapor, thereby inducing a small amount of physical volatilization behavior [48,49]. Moreover, in the temperature range of 200 to 500 °C, cellulose, hemicellulose, and lignin, along with other significant organic components, undergo vigorous thermal decomposition. Given that this process occurs under these conditions, this stage is characterized by the release of volatile matter and the subsequent combustion of fixed carbon. Nevertheless, as the heating rate increases, the TG and DTG curves shift collectively toward higher temperatures. Thus, the main characteristic temperatures (e.g., *T_i_, T_b_,* and *T_f_*) and their corresponding time points (*t_i_* and *t_b_*) show a significant increase. However, the TG curve exhibits two distinct downward trends, while the DTG curve displays two prominent peaks with a gradual transition toward peak merging. This is consistent with the results of the volatile matter release and fixed carbon combustion segmentation in Reference [50].

The analysis suggests that the main DTG peak shifts to a lower temperature under oxy-fuel combustion conditions compared to air, and the peak shape becomes sharper, indicating that the primary combustion reactions are more concentrated and intense. Under 30% O_2_ conditions, the elevated CO_2_ concentration acts as a thermal and chemical diluent, suppressing the volatile release through reduced partial pressure and attenuated heat transfer. However, in a 70% O_2_ environment, these inhibitory effects are likely overshadowed by the dominance of intense oxidation reactions, resulting in a significantly higher combustion efficiency relative to air atmospheres. As shown in Table 4, as the O_2_ concentration increases from 30% to 70%, both the ignition temperature (*T_ᵢ_*) and the burnout temperature (*T_b_*) of RS decrease significantly. At 70% O_2_, ignition temperatures under the three heating rates decrease by 27 °C, 26 °C, and 28 °C, respectively, while burnout temperatures decrease by 135.5 °C, 152 °C, and 40 °C. Furthermore, the data clearly indicate that increasing the heating rate and elevating the O_2_ concentration both contribute to shorter ignition and burnout times, demonstrating a substantial acceleration of the combustion rate. Especially when the O_2_ concentration reaches 70%, the maximum mass loss rate (dw/dt)_max_ is far higher than the value in an air atmosphere, indicating an extremely intense reaction process.

The combustion index (*S*) exhibits substantial increases, indicating a marked enhancement in combustion intensity, which is a phenomenon of considerable significance. Moreover, at the higher heating rate of 30 °C/min, the combustion rate increases further, reaching more than tenfold that observed in air under 70% O_2_. In contrast, at the lower heating rate of 10 °C/min, *t_max_* is prolonged with increasing O_2_ concentration. This occurs because, under milder heating conditions, the oxy-fuel atmosphere accelerates the reaction kinetics and promotes the more complete release and combustion of volatile matter, thereby delaying the time at which the peak reaction rate is attained. Conversely, at higher heating rates (e.g., 20 and 30 °C/min), *t_max_* decreases with increasing O_2_ concentration. These results indicate that rapid heating synergizes with a high O_2_ concentration and elevated temperature to promote a rapid transition of the fuel into a vigorous combustion state, as evidenced by an earlier onset and increased intensity of the combustion peak. Collectively, the pronounced variation in t_max_ reveals a critical interactive effect between the O_2_ concentration and heating rate on the overall combustion behavior.

At all the experimental heating rates, the absolute value of *C_i_* significantly increased with the increase in oxygen concentration. It is noteworthy that, at a heating rate of 10 °C/min, the absolute value of *C_i_* in a 70% oxygen environment was approximately 8 times higher than that in an air atmosphere, while, at 30 °C/min, this increase reached 14 times. The significant growth of the absolute value of *C_i_* directly reflects the enhancement in ignition performance. This is because the high-oxygen-concentration environment promotes the release of volatile matter and the initial oxidation reaction, reducing the activation energy required for ignition, thereby significantly improving the combustibility of the fuel. The experimental results clearly confirm that oxygen-rich combustion can significantly improve the combustion start-up characteristics. Moreover, consistent with the trend of *C_i_* changes, the absolute value of *C_b_* also increased dramatically under different experimental conditions. For example, at a heating rate of 20 °C/min, the absolute value of *C_b_* in a 70% oxygen environment increased by more than 10 times compared to the air atmosphere. The increase in *C_b_* value indicates that the combustion rate has been optimized, which is highly consistent with the thermal analysis conclusion in Reference [51]. In summary, the oxygen-rich atmosphere accelerates the combustion of volatile matter in the early stage of combustion and strengthens the oxidation of fixed carbon in the later stage, enabling the residual coke to be completely consumed at a lower temperature and shorter time (which is consistent with the observed decrease in *T_b_*). Therefore, oxygen-rich combustion can effectively reduce incomplete combustion losses and the unburned carbon content, significantly improving the fuel utilization efficiency.

**Figure 5:**
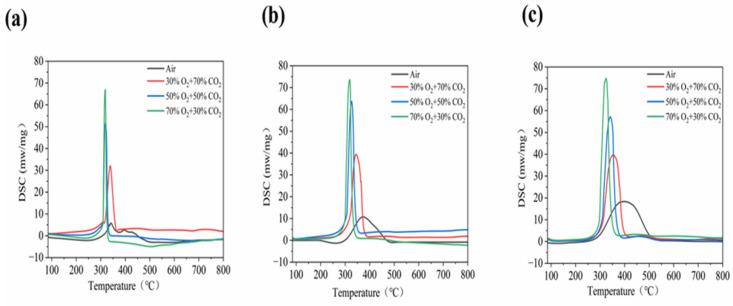
DSC curves of different reaction atmospheres under different temperature conditions: (**a**–**c**) represent the DSC changes at 10, 20, and 30 °C/min, respectively.

Oxy-fuel combustion accelerates the combustion reaction kinetics of biomass and enhances the overall heat release during combustion. The influence of oxy-fuel combustion on the heat release behavior of RS across different heating rates is predominantly observed during the stages of volatile release and combustion. The DSC curve illustrates the variation pattern of the heat released by RS throughout the entire combustion process under different atmospheres as a function of the temperature and heating rate. The corresponding heat flow profiles are presented in Figure 5. As the O2 concentration in the oxy-fuel atmosphere increases, the combustion peak shape transitions from broad to narrow. The research findings indicate that, under different heating rates, the first peak corresponding to the combustion of volatile matter in the DSC curve is consistently higher than the second peak corresponding to the combustion of fixed carbon. This difference stems from the disparities in the reaction mechanisms and energy release characteristics of the two. The relatively high calorific value of volatile matter is primarily attributed to its rich hydrogen content. The combustion heat per unit mass of hydrogen is approximately four times that of carbon, which makes a significant contribution to the overall energy intensity of volatile matter.

Volatile matter, which accounts for 50% to 70% of the total energy content of the fuel, primarily consists of light hydrocarbons, CO, and H_2_. These components undergo rapid and complete gas-phase oxidation, leading to a concentrated heat release that manifests as a sharp, high-intensity exothermic peak in the DSC curve. In contrast, fixed carbon combustion involves solid gas heterogeneous reactions governed by O_2_ diffusion to the carbon surface, resulting in a broader and more moderately intense peak. This process is often constrained by kinetic and physical limitations such as ash encapsulation or incomplete oxidation to CO, which reduce the energy release rate compared to volatile matter within a given timeframe. When the O_2_ concentration is increased from 50% to 70%, these dynamics are significantly amplified, causing the first exothermic peak to become markedly more intense than the second. The enhanced O_2_ diffusion driving force accelerates the fixed carbon combustion, leading to a narrower and left-shifted peak that indicates an improved low-temperature reactivity. Despite this acceleration, the rapid kinetics and high calorific value of the volatile matter ensure its heat release peak remains dominant. This intensified combustion behavior in oxy-fuel environments aligns with the statistical findings for various fuels including hard coal and biomass [52].

Most studies on the oxy-fuel combustion of biomass only focus on the chemical and physical levels, but ignore the coupling effect between them. The strong thermal inertia brought about by thermodynamic characteristics such as the high density(*ρ*) of CO_2_ and high specific heat capacity (*C_p_*) mainly affects the temperature response sensitivity and the form of exothermic signals of the curve. The CO_2_ atmosphere heats up more slowly under the same heat input, causing the temperature rise within the fuel particles to lag behind the environment. This is manifested as a slight inhibition of the volatile component release period at low oxygen concentrations (such as 30% O_2_), and a slight rightward shift of the DTG curve’s starting point due to the thermal buffering effect. The higher heat capacity (*C_p_*) reduces the temperature fluctuations in the flame and reaction zone, reflected in the DTG curve as a more stable combustion process with smaller peak fluctuations. The large molecular weight and multiple vibration degrees of freedom of CO_2_ enable it to absorb more reaction latent heat, making the thermal flow signal detected by DSC more “sluggish” compared to the N_2_ atmosphere. As the oxygen concentration increases, although the reaction intensifies, the thermal buffering effect of CO_2_ causes the exothermic peaks of the volatile matter and fixed carbon to merge more on the temperature axis, and the curve shape changes from broad to sharp, reflecting the transformation from the stepwise to synchronous strengthening of the reaction.

The shift in the TG/DTG peak positions is quantitatively driven by the evolution of thermal physical properties. This study deeply analyzed the underlying “physical–dynamic” coupling mechanism: as the oxygen concentration increased from 30% to 70%, the heat transfer characteristics of the mixed gas underwent a significant quantitative improvement. At the reaction zone of 800 K, the thermal diffusivity a increased by 14.4%, effectively reducing the heat transfer thermal resistance at the gas–solid interface; meanwhile, the gas density ρ decreased by 11.7%, significantly weakening the thermal inertia effect caused by the high concentration of CO_2_. The analysis of dimensionless numbers indicated that the Pr rose by approximately 2.4% with the increase in oxygen concentration, reflecting the fine regulation of the ratio of the momentum and heat diffusion. This optimization of physical properties quantitatively reduced the thermal lag effect, combined with the kinetic acceleration brought about by the increase in the oxygen partial pressure, jointly driving the shift of the TG/DTG peak position towards the low-temperature region. The experimental results quantitatively confirmed that, for every 10% increase in oxygen concentration, the peak position shifted, on average, by approximately 10 °C. In conclusion, through the quantitative correlation of the Pr and key thermal physical parameters, it was revealed that the shift in the TG/DTG peak positions is not merely a chemical kinetic response, but a macroscopic manifestation of the deep coupling between the “physical thermal delay” caused by CO_2_ and the enhancement in oxygen diffusion.

The exothermic peaks in the DSC curves align closely with the corresponding mass loss rate peaks in the DTG curves across various conditions, providing a robust validation of the RS combustion consumption data. However, the temperature disparity between these signals increases with the heating rate, a phenomenon primarily attributed to the pore structure of RS particles and the resulting modulation of temperature gradients at the gas–solid interface. This thermal lag is further intensified by the transport characteristics of CO_2_, including its low thermal conductivity (*k*), low thermal diffusivity (*a*), and reduced diffusion rate of oxygen within this atmosphere. These thermophysical limitations increase the resistance to oxygen diffusion into the particles, thereby restricting the macroscopic chemical reaction rate. Although high oxygen concentrations typically shift the main DTG peak toward lower temperatures, the low diffusivity of CO_2_ effectively slows this trend. Consequently, an increased heating rate exacerbates the mass transfer resistance and hinders the gas–solid energy transfer, shifting T_max_ to higher temperatures and widening the lag between the DSC and DTG signals. This process culminates in a physical decoupling where the chemical reaction rate and macroscopic heat release no longer align in terms of temperature and time, which is consistent with the findings in Section 3.1.

The difference between this oxy-fuel combustion and traditional kinetic studies lies in that it integrates, for the first time, the thermal physical transport characteristics of the atmosphere (such as the thermal diffusivity, momentum diffusion, and Pr) with multi-dimensional TG-DSC-MS data in depth. This approach clarifies how the high thermal inertia and low diffusivity of CO_2_ intervene in the combustion process by altering the thermal mass transfer boundary layer, revealing the coupling mechanism between the physical environment and chemical kinetics. This breaks through the limitation of previous studies that only focused on the chemical reaction rate, providing a more comprehensive perspective for understanding oxy-fuel combustion.

**Figure 6:**
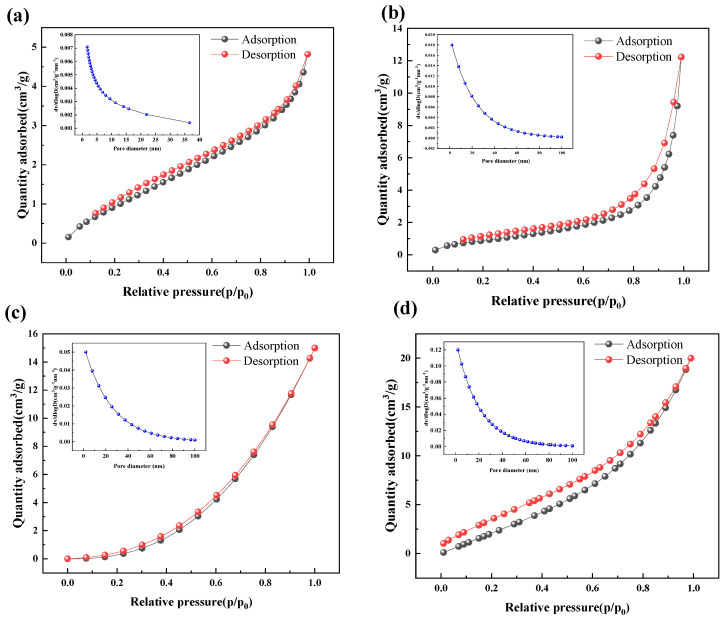
N_2_ adsorption–desorption curves and particle size distribution diagrams of combustion products under different reaction atmospheres at 800 °C: (**a**–**d**) represent the BET changes of Air, 30% O_2_/70% CO_2_, 50% O_2_/50% CO_2_, and 70% O_2_/30% CO_2_, respectively. Insets (blue dots) show high-intensity micro/small mesopore distributions across all atmospheres.

Figure 6 illustrates the nitrogen adsorption and desorption isotherms and the pore size distribution profiles of the RS combustion products generated under various reaction atmospheres at 800 °C. By comparing Figure 6a to Figure 6d, it can be observed that the maximum adsorption capacity of the combustion products in the air atmosphere is the lowest (approximately 5 cm^3^/g). From Figure 6b to Figure 6d, it can be observed that, in an oxy-fuel environment, as the oxygen concentration increases from 30% to 70%, the adsorption capacity shows a significant growth trend. Under the condition of 70% O_2_/30% CO_2_, the maximum adsorption capacity reached approximately 20 cm^3^/g. This indicates that, in an oxy-fuel environment, especially under conditions of a high oxygen concentration, the combustion reaction becomes more intense and complete, which is more conducive to the formation and opening of the internal pore structure of the fuel. Oxy-fuel combustion significantly increased the combustion rate of RS. At a heating rate of 30 °C/min and under 70% O_2_ conditions, the combustion rate exceeds 10 times that of the air. This intense reaction process greatly promoted the efficient release of volatile matter and the deep oxidation of fixed carbon. As shown in Figure 6, the increase in the adsorption amount of the combustion product N_2_ quantitatively confirms the drastic evolution of the adsorption intensity at the active sites. This significant enhancement in adsorption capacity reflects the more thorough consumption and transformation of the organic components in the fuel, thereby forming an inorganic ash skeleton or activated carbon structure with high chemical activity.

The inset figures (blue dots) in each atmosphere all show that there is an extremely high distribution intensity within the small pore size range (micro pores/small mesopores). As the oxygen concentration increases, the peak values of these small pore diameters rise significantly. This indicates that the oxy-fuel environment not only increases the total pore volume but also significantly enhances the specific surface area. This is consistent with the conclusions mentioned in Table 4, namely, “improving fuel utilization efficiency” and “reducing unburned carbon content”. The analysis in Figure 6 provides crucial physical evidence for the article: it demonstrates that the CO_2_ environment, combined with a high concentration of O_2_, can overcome its own high thermal inertia and low diffusion rate limitations. By intensifying the oxidation reaction, a more thorough material transformation than traditional air combustion was achieved, thereby generating combustion products with a larger specific surface area and a more developed pore structure.

### 3.3. Gas Emission Characteristics of RS

During the RS combustion process, the release intensities of different pollutant gases do not represent independent events but rather reflect integrated outcomes resulting from the combined influence of key atmospheric composition factors and heating rate conditions. This synergistic regulation arises from the interplay between the thermodynamic properties of the system and the heat and mass transfer characteristics of the gaseous environment. This section focuses on the emission behavior of NO_X_, SO_2_, and COS. Under rapid heating in an oxy-fuel atmosphere, the combination of a high temperature and an elevated O_2_ concentration promotes the efficient conversion of fuel-bound N_2_ into NO and NO_2_. Concurrently, a high heat flux enhances sulfur release, predominantly in the form of SO_2_. In contrast, under O_2_-deficient or slow-heating conditions, pyrolysis becomes the dominant reaction pathway, which suppresses NO_X_ and favors the formation of COS as the primary sulfur species, while a portion of sulfur remains retained in the char matrix [42], thereby reducing the immediate release of SO_2_.

**Figure 7:**
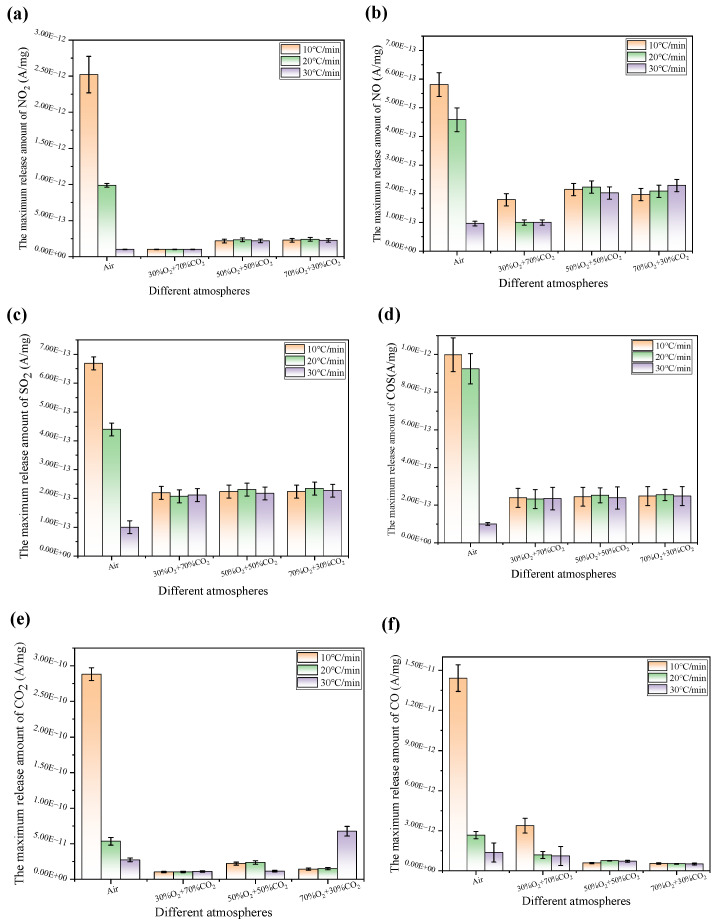
Gas release characteristics under different reaction atmospheres at different heating rates: (**a**–**f**) represent the intensity characteristics of the maximum release amounts of NO_2_, NO, SO_2_, COS, CO_2_, and CO, as determined through real-time monitoring of characteristic mass-to-charge ratios (*m*/*z* = 46, 30, 64, 60, 44, and 28) via a triple quadrupole mass spectrometer and thermocouple. The length of the error bars indicates the magnitude of the error.

As shown in Figure 7, NO_2_ emissions in air are approximately ten times higher than those in the oxy-fuel atmosphere. This phenomenon primarily stems from the relatively high thermal diffusivity of N_2_ in air (21% O_2_/79% N_2_) (*α* is approximately 1.83 × 10^−4^ m^2^/s at 800 °C), which facilitates the processes of oxygen diffusion and NO_2_ generation reactions. In the oxygen-enriched combustion atmosphere with CO_2_ as the carrier gas, CO_2_ exhibits a relatively high density (ρ is approximately 0.5 kg/m^3^ at 800 °C) and a large heat capacity (*C_v_* is approximately 1.064 kJ/(kg·K) at 800 °C and *C_p_* is approximately 1.253 kJ/(kg·K) at 800 °C). These properties lead to a significant increase in thermal inertia. Moreover, the diffusion rate of O_2_ in CO_2_ is relatively low. Collectively, these factors inhibit the generation of NO_2_, resulting in a release intensity far lower than that in the air atmosphere. In light of the elevated O_2_ concentration in a 30% O_2_/70% CO_2_ atmosphere, the thermal diffusivity of CO_2_ appears approximately 29.95% lower than that of N_2_. Thus, CO_2_ exhibits a density about 1.57 times higher and a specific heat capacity approximately 1.11 times greater than those of N_2_; these physical properties indicate CO_2_ possesses a significantly higher thermal inertia and lower thermal diffusivity.

As shown in Figure 7a, these significant characteristics collectively govern the critical heat and mass transfer processes within the reaction system, thereby leading to the consistently low NO_2_ release intensity that appears evident. Under air conditions, NO_2_ emissions decrease with increasing heating rate. In contrast, under oxy-fuel conditions, NO_2_ release follows a three-stage trend—an initial gradual increase followed by stabilization—as the heating rate increases from 10 °C/min to 20 °C/min and 30 °C/min, with the overall increase not exceeding 10%. However, a higher heating rate enhances the oxidation reaction kinetics. Nevertheless, the high heat capacity of CO_2_ induces a lag in the system temperature response, which counteracts promoting the effect of the increased heating rate. Thus, NO_2_ release profiles across different heating rates exhibit similar shapes, with minimal variation in the peak emission levels. In summary, the important NO_2_ release is governed by the synergistic influence of the thermal properties (high C_v_, high ρ, and low α) and heating rate, with these significant factors collectively suppressing NO_2_ formation by more than 90%.

As shown in Figure 7b, the release behavior of NO across different atmospheres indicates that the regulatory mechanism appears to be fundamentally similar to that observed for NO_2_, given that both significant chemical species are synergistically influenced by the critical atmospheric physical properties and the substantial heating rate. In an air atmosphere, the important amount of NO released demonstrates a decreasing trend with increasing heating rate. Moreover, the low thermal inertia of N_2_ attenuates the thermal lag effect. Thus, the system temperature responds to the heating program. Increasing the heating rate reduces the residence time of the reaction system within the high-temperature zone necessary for the efficient formation of thermal NO. Furthermore, the generation of thermal NO depends on the cumulative exposure to high temperature and time duration. This reduction in effective reaction time results in a lower total emission. Nevertheless, under an oxy-fuel atmosphere, the significant release behavior exhibits distinct variations with respect to the critical changes in O_2_ concentration and heating rate. At a heating rate of 10 °C/min, the important NO release amount demonstrates a decreasing trend. However, at 20 °C/min, it initially increased, followed by a decrease. At 30 °C/min, an increasing trend was observed. In light of these experimental findings, the NO release reaches its minimum level under a 30% O_2_/70% CO_2_ atmosphere at heating rates of 20 °C/min and 30 °C/min. The thermal NO formation pathway was substantially suppressed, leading to an approximately 75% reduction in total NO emissions. This suppression is primarily attributed to the increasing heating rate, which causes the internal particle temperature to lag significantly behind the furnace temperature, thereby promoting incomplete reactions. In high-O_2_-concentration environments, NO is more readily oxidized to NO_2_. Therefore, for power plants employing oxy-fuel combustion under high-heating-rate conditions, the implementation of staged combustion or flue gas recirculation is recommended to strengthen the reducing environment within the high-temperature zone and mitigate the continuous accumulation of NO.

As shown in Figure 7c, under air atmosphere conditions, SO_2_ emissions decrease with increasing heating rate. In an oxy-fuel atmosphere, SO_2_ emissions remain essentially stable at a heating rate of 10 °C/min, gradually increase at 20 °C/min, and stabilize again at 30 °C/min. Overall, the lowest SO_2_ emissions occur under air atmosphere conditions at a heating rate of 30 °C/min, resulting in minimal environmental impact. The emission amount decreases by approximately 65% at this condition, primarily because the high heating rate suppresses the low-temperature decomposition and medium-temperature oxidation of sulfates. Under oxy-fuel conditions, SO_2_ emissions exhibit little variation across different heating rates. Across varying atmospheric compositions and heating rates, the SO_2_ release behavior is predominantly governed by the physical properties of the gaseous environment and associated mass transfer processes. Compared to N_2_, CO_2_ has a higher density and greater heat capacity (C_p_ exceeding that of N_2_ at 400 °C), but its diffusion coefficient is only about 70% of that in N_2_, and its thermal conductivity is reduced by 3%. These differences lead to a restricted O_2_ transport and reduced heat transfer efficiency during combustion in an oxy-fuel environment.

As shown in Figure 7d, the sulfur present in fuel reacts with the carbon monoxide that is generated during incomplete combustion processes to form the compound carbonyl sulfide (COS). In light of humid environmental conditions, COS emissions undergo significant hydrolysis reactions to produce H_2_S and CO_2_, both of which appear to be irritants to the respiratory tract [43]. Moreover, research on COS emissions is essential, particularly when these emissions are compared to studies of conventional pollutants. The release of COS in an air atmosphere decreases with increasing heating rate. At a heating rate of 30 °C/min, COS emissions reach their minimum level, exhibiting an approximate 80% reduction. Under an oxy-fuel atmosphere, as the heating rate increased from 10 °C/min to 30 °C/min, the release of COS exhibits minimal variation. Furthermore, COS emissions remain stable at 10 °C/min, show only a slight increase at 20 °C/min, and persist at a relatively low level at 30 °C/min. Given that the mass diffusion rate of O_2_ in a CO_2_ environment under oxy-fuel conditions appears relatively low, this phenomenon arises primarily from these diffusion limitations. Thus, as the heating rate increases, the surface reaction accelerates; however, the inward diffusion of O_2_ does not keep pace, resulting in a lack of synchronization between the reaction kinetics and mass transport. Therefore, the internal O_2_ partial pressure remains relatively low, and the overall reaction continues to be governed by diffusion kinetics, thereby reducing the influence of the elevated heating rate. It is consistent with the comprehensive assessment of sulfur oxide and nitrogen oxide emission reduction introduced in Reference [53].

Figure 7e illustrates the variation pattern of the maximum CO_2_ release intensity. Under an air atmosphere, the CO_2_ release intensity reaches its peak at a lower heating rate (10 °C/min) and shows a significant downward trend as the heating rate increases (20 and 30 °C/min). In contrast, the CO_2_ release intensity in an oxygen-rich atmosphere is significantly lower than that in the air atmosphere at a heating rate of 10 °C/min. However, under oxygen-rich conditions, this intensity exhibits a clear positive correlation: as the oxygen concentration (ranging from 30% to 70%) and the heating rate increase simultaneously, the maximum release intensity of CO_2_ significantly increases. This phenomenon validates the “synergistic enhancement effect” described in the text; that is, the high oxygen concentration and the high heating rate jointly promoted the rapid transition of the fuel to the intense combustion stage, making the reaction more concentrated and intense, thereby significantly enhancing the instantaneous release intensity of CO_2_.

Figure 7f illustrates the maximum release intensity of CO under different atmospheres. As the incomplete combustion products generated when the fuel is in an oxygen-deficient environment or the reaction is insufficient, the release amount of CO is a key indicator for measuring the combustion efficiency. The experimental comparison revealed that the air atmosphere produced the highest intensity of CO release at a low heating rate; in contrast, the CO release in all oxygen-rich atmospheres was significantly lower than that in air. The oxygen concentration played a decisive role in this: as the O_2_ concentration increased from 30% to 70%, the release intensity of CO continued to decrease, and, at a concentration of 70%, it was almost undetectable. This result strongly validates the conclusion of this paper, which is that oxygen-rich combustion significantly reduces incomplete combustion losses by enhancing the driving force for oxygen to diffuse towards the carbon surface. By optimizing the reaction kinetics, the oxygen-rich environment (especially with a high oxygen concentration) enables the carbon element to be more completely and concentratedly converted into CO_2_. This not only enhances the efficiency of energy release but also effectively inhibits the generation of harmful gas CO.

**Figure 8:**
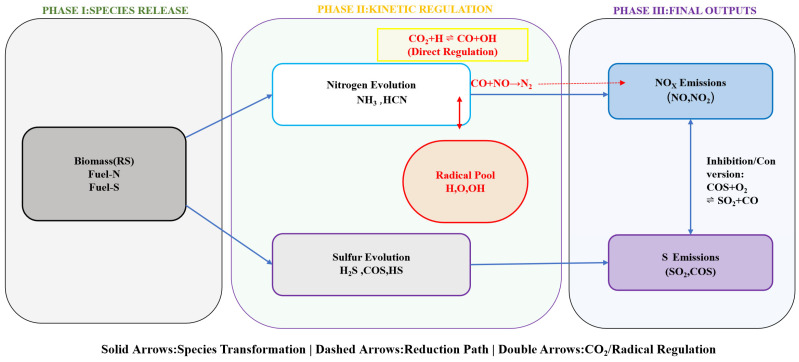
The synergistic transformation and regulatory mechanism of typical gaseous pollutants (NO_2_, NO, SO_2_, COS, CO_2_, and CO) in an oxy-fuel combustion of RS.

Figure 8 provides a detailed illustration of the synergistic transformation and regulation mechanism of gaseous pollutants during the combustion process of RS in an oxy-fuel combustion. The entire process can be divided into three key stages: the species release stage, the kinetic regulation stage, and the final output stage. In the first stage, the species release (Phase I Species Release) process begins with biomass (RS) particles containing fuel-type nitrogen (Fuel-N) and fuel-type sulfur (Fuel-S). As the pyrolysis and combustion commence, the N and S elements within the fuel start to transform into the gaseous phase.

As the core characteristic that distinguishes oxygen-enriched combustion from air combustion, the second stage of kinetic regulation involves a series of complex free radical reactions. Firstly, CO_2_ directly participates in the regulatory process. High concentrations of CO_2_ consume H free radicals through the elementary reaction CO_2_ + H = CO + OH and generate OH free radicals, thereby significantly reshaping the composition ratio of the free radical pool. On this basis, the evolution paths of nitrogen and sulfur elements change: when the fuel nitrogen is converted into intermediate products NH_3_ and HCN, it is affected by the bidirectional regulation of the free radical pool (as indicated by the red double arrows in the Figure 8), determining the final direction of its conversion to NOx; at the same time, the fuel sulfur is converted into precursors such as H_2_S, COS, and HS. Moreover, the reduction path (Reduction Path) is also crucial in this stage, as indicated by the dotted arrow in the figure. In an oxygen-rich environment, by using means such as staged combustion to enhance the local reduction atmosphere, the reaction between CO and NO can be effectively promoted, converting the generated NO into harmless N_2_.

In the Phase III Final Outputs of the third stage, NO_x_ emissions are mainly composed of NO and NO_2_. Research indicates that, due to the high heat capacity and low diffusion rate of CO_2_, an oxygen-rich environment can effectively inhibit the formation of thermal NO, thereby significantly reducing the NO_x_ emissions. In terms of sulfur (S) emissions, the final products mainly include SO_2_ and COS. The conversion relationship shown in Figure 8, COS + O_2_ = SO_2_ + CO, reveals its inhibition and transformation mechanism: in an environment with a higher oxygen concentration, the equilibrium shifts towards the generation of SO_2_, while, in local hypoxic or high-heating-rate conditions, affected by diffusion limitations, COS becomes the main emission. This flowchart systematically reveals how an oxygen-rich environment, by changing the physical and chemical properties of the atmosphere (such as a high CO_2_ concentration and radical regulation), comprehensively reduces pollutant generation from the source of precursor formation to the end of the reduction path. This synergistic regulatory mechanism provides an important theoretical basis for the clean and efficient utilization of biomass. It is consistent with the inhibition mechanism of sulfur and nitrogen oxides described in References [54,55].

Fuel-derived NO_x_ originates from nitrogen-containing precursors such as NH_3_ and HCN that are released during the thermal decomposition of Fuel-N in straw. CO_2_ exerts a profound regulatory influence on fuel-derived NO_x_ formation by modulating the concentrations and reactivities of key free-radical intermediates within the reaction network, thereby shaping the overall NO_x_ generation profile. In contrast, thermal NO_x_ formation is markedly suppressed due to the CO_2_ high thermal inertia, which induces a “thermal lag” effect and reduces the residence time of combustion gases in the high-temperature regime. Concurrently, Fuel-S is thermally decomposed to yield gaseous precursors including H_2_S and COS, which subsequently undergo oxidative conversion via the oxygen-concentration-dependent reaction COS + O_2_ = SO_2_ + CO, ultimately establishing a dynamic equilibrium among sulfur-containing species.

This study systematically analyzed the co-conversion and regulation mechanisms of typical gaseous pollutants NO_x_, SO_2_, and the less-studied COS in oxy-fuel combustion. It particularly revealed that high-concentration CO_2_ directly reshaped free radicals by participating in the elementary reaction CO_2_ + H = CO + OH, thereby establishing a kinetic intervention strategy for the generation of precursors. Compared with previous studies that were limited to the description of emission characteristics, this paper achieved a full-pathway analysis from species release to final output.

### 3.4. Combustion Reaction Kinetics

#### 3.4.1. Kinetic Parameters of Chemical Reactions

**Figure 9:**
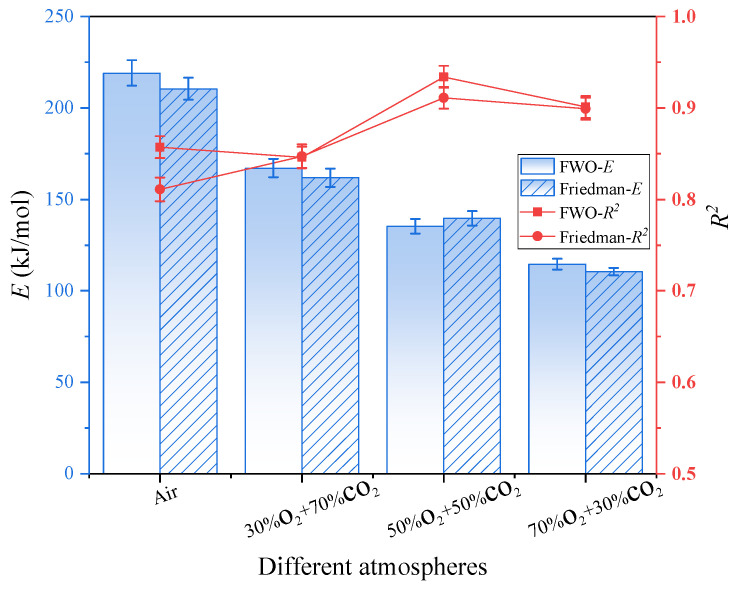
Comparison of activation energy (*E*) and coefficient of determination (*R*^2^) values under different reaction atmospheres by FWO and Friedman methods. The length of the error bars represents the magnitude of the error.

The distribution of activation energy *E* and the correlation coefficient *R*^2^ as a function of increasing O_2_ concentration are determined using the FWO and Friedman methods, thereby enabling a significant kinetic analysis of the entire combustion process [56]. Moreover, as illustrated in Figure 9, when the O_2_ concentration increased from 21% to 70%, the activation energy values derived from the FWO and Friedman methods have exhibited a decreasing trend, declining from 210.5 kJ/mol to 110.5 kJ/mol and from 219.1 kJ/mol to 114.6 kJ/mol, respectively, representing reductions of 47.5% and 47.7%. This is consistent with the gas pressurization and oxidation baking methods for the pyrolysis of RS, such as those proposed by FWO and Friedman [57]. This indicates that, under low O_2_ or normal air conditions, the combustion reaction must overcome a relatively high energy barrier, resulting in a more difficult reaction progression. However, under high O_2_ conditions, the combustion process is enhanced, and the activation energy required for the reaction is reduced. Nevertheless, the primary reason appears to be that a high O_2_ concentration enhances the effective collision frequency between O_2_ molecules and fuel, thereby accelerating the reaction rate. Given that an oxy-fuel environment promotes the formation and transfer of reactive radicals (such as·O and ·OH), this facilitates the progression of chain reactions [58]. Furthermore, as the concentration of O_2_ increases, the proportion of CO_2_ serving as a diluent decreases correspondingly, thereby reducing its inhibitory effect on the combustion reaction.

**Figure 10:**
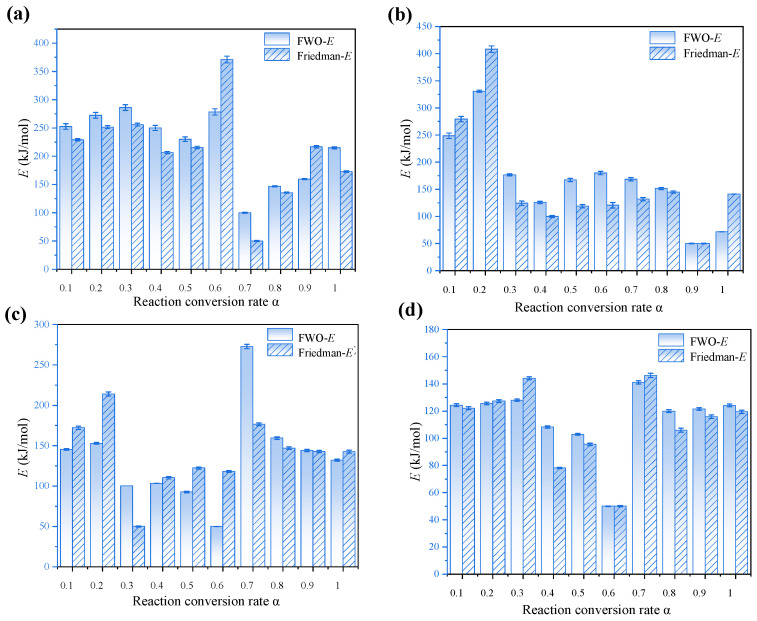
Comparison of activation energies calculated by FWO and Friedman methods across different conversion degrees α: (**a**–**d**), respectively, represent the changes in activation energy for Air, 30% O_2_/70% CO_2_, 50% O_2_/50% CO_2_, and 70% O_2_/30% CO_2_.

The error bars associated with the activation energy values presented in Figure 9 and Figure 10 exhibit systematic variation depending on both atmospheric composition and conversion degree (α). Specifically, as the oxygen concentration increases from 30% to 70%, the magnitude of the error bars consistently decreases. This trend reflects a simplification of the underlying reaction mechanism under elevated oxygen partial pressure, leading to a more uniform and localized energy release—thereby reducing experimental uncertainty. Furthermore, the activation energy values demonstrate high reproducibility across three independent replicate experiments, indicating robust experimental consistency and data stability. As illustrated in Figure 9, the *R*^2^ values obtained from the FWO and Friedman methods remain high overall, generally exceeding 0.8. This indicates that both methods exhibit a strong linear fitting performance and that the corresponding kinetic models are highly applicable to the combustion process. Thus, the R^2^ value exhibits minimal variation across different O_2_ concentrations, indicating both methods maintain stability and reliability under varying atmospheric conditions.

The kinetic analysis of oxy-fuel combustion processes involving lignocellulosic materials such as RS indicates that notable differences appear between the FWO method and the Friedman method. Moreover, lignocellulose constitutes only 75.98% of the sample, and the oxy-fuel combustion process demonstrates distinct kinetic behavior. Furthermore, the relatively simplified gas atmosphere, narrow reaction temperature range, and simple compositional structure influence this behavior. Given that the FWO method is based on the integral approach, the method assumes a constant activation energy. Thus, the method is applied by analyzing relationships between the heating rate and temperature. However, the integral smoothing effect reduces the noise interference from combustion fluctuations. Additionally, the method appears well-suited for characterizing the overall combustion behavior of the system. Nevertheless, the underlying reaction mechanism is gradual and consistent with kinetic stability. In contrast, the Friedman method is based on differential methods, directly solving for the instantaneous relationship between the reaction rate and temperature without presupposing a constant activation energy, making it more sensitive to changes in the reaction process. However, in the relatively homogeneous process of the oxy-fuel combustion of lignocellulose, the characteristic of the differential method to amplify noise significantly affects the results due to experimental fluctuations. Moreover, this system rarely exhibits complex multi-stage reaction pathway changes, so the dynamic analysis advantage of the Friedman method is not prominent in such scenarios [59,60,61,62]. In conclusion, the FWO method is generally well-suited for the kinetic analysis of lignocellulosic materials such as RS under oxy-fuel combustion conditions, offering a favorable balance between computational stability and result reliability. As shown in the error bars of Figure 9 and Figure 10, when the FWO method is used to process the combustion data of RS, a type of multi-component complex material, it usually exhibits a smaller fluctuation range and higher numerical stability compared to the Friedman method. This is consistent with the characteristic explanations of FWO and the Friedman method mentioned in Reference [62].

In the TGA, the complex process involving water loss, volatile component release, and fixed carbon combustion, which consists of multiple overlapping stages, is treated as a “kinetic homogeneous” process. Although the combustion of RS is theoretically divided into multiple stages, the experimental data show that, in an oxy-fuel combustion, especially when the oxygen concentration is increased to 50–70%, the release of volatile components and the combustion of fixed carbon occur significantly overlapping in time and space. As mentioned in Section 3.1 of the previous part, as the oxygen concentration increases, the heat release peaks of volatile components and fixed carbon on the DSC curve gradually approach and tend to merge, and the reaction shifts from “stepwise” to “simultaneous” progress. This synchronization phenomenon indicates that, in a high-intensity oxidation environment, the kinetic boundaries of each stage become blurred, and the entire combustion process macroscopically appears as a concentrated intense heat release event, allowing the use of global kinetic parameters for the description. At a high heating rate (20–30 °C/min), the temperature gradients inside and outside the particles and the thermal lag effect further strengthen the “global synchronization” feature of the reaction, making it more in line with the theoretical assumptions of the standard mechanism function. From the perspective of material composition, the composition of RS is relatively simple, as the dominant component is lignocellulose, and its reaction mechanism during the combustion process is relatively gentle and continuous, providing a material basis for obtaining a single average activation energy (*E*). As mentioned in the following Section 3.4.2, under the coupling conditions of a high oxygen concentration and high heating rate, the reaction mechanism will shift from complex diffusion control to a more unified random nucleation and growth model (Avrami–Erofeev equation), and this dynamic simplification of the mechanism further demonstrates the scientific nature of treating it as a kinetic homogeneous process.

Figure 9 shows the evolution law of activation energy with respect to α under four different reaction atmospheres, calculated using the FWO method (integral method) and the Friedman method (differential method). By comparing the four sub-figures, it can be clearly observed that the activation energy decreases significantly with the increase in oxygen concentration, indicating that the increase in oxygen content effectively reduces the reaction barrier, thereby enhancing the reaction activity. Under the air atmosphere, the reaction exhibited the highest activation energy, with its value remaining at 250 kJ/mol throughout most of the α range. In contrast, in the oxy-fuel combustion, the activation energy showed a significant decreasing trend as the O_2_ concentration increased from 30% to 70%. Particularly in the 70% O_2_ atmosphere, the activation energy reached its lowest value (approximately 100–140 kJ/mol).

Based on the dynamic thermal analysis and stage-wise characterization, a conversion degree α = 0.5 is identified as the critical threshold delineating the two principal combustion stages: volatile matter release and fixed carbon oxidation. Specifically, Combustion Stage I (α < 0.5) encompasses the initial thermal decomposition of biomass constituents, namely, hemicellulose, cellulose, and lignin, followed by vigorous gas-phase oxidation of the evolved volatiles. As illustrated in Figure 4, this stage is characterized by a pronounced volatile release peak centered approximately within the temperature range of 280–320 °C. In contrast, Combustion Stage II (α > 0.5) corresponds to the heterogeneous oxidation of residual char, predominantly occurring between 320–450 °C. Under elevated heating rates (20–30 °C/min) and high oxygen concentrations (50–70%), the exothermic peaks associated with volatile release and char combustion increasingly overlap. Consequently, the kinetic distinction between the two stages diminishes, resulting in a macroscopically unified, intense exothermic event.

Figure 10 presents a comparative analysis of *E* derived from the FWO and Friedman methods across a range of conversion degrees (α). The Friedman method—depicted as the shaded region—exhibits a pronounced variability in *E* attributable to its intrinsic sensitivity to experimental noise, as it relies on the numerical differentiation of thermogravimetric data. In contrast, the integral-based FWO method demonstrates a superior smoothing capability and computational robustness, rendering it more appropriate for elucidating the multi-stage, heterogeneous combustion kinetics of lignocellulosic residual solids. The systematic variation of *E* with α reflects the mechanistic complexity of biomass combustion, which involves concurrently occurring and interdependent processes—including volatile release, secondary cracking, and char oxidation. Notably, under a 70% O_2_ atmosphere, E converges to an asymptotic plateau at a higher α, suggesting that an elevated oxygen concentration homogenizes the reaction pathway and facilitates a more consistent and energetically focused combustion. The lower activation energy means that the effective collision frequency between oxygen molecules and fuel increases, making it easier for the reaction to overcome the energy barrier. The enriched oxygen environment promotes the formation and transfer of active radicals (such as ·O and ·OH), thereby accelerating the progress of the chain reaction. The increase in oxygen concentration also means that the proportion of dilute gas CO_2_ decreases, weakening its inhibitory effect on the combustion reaction.

**Figure 11:**
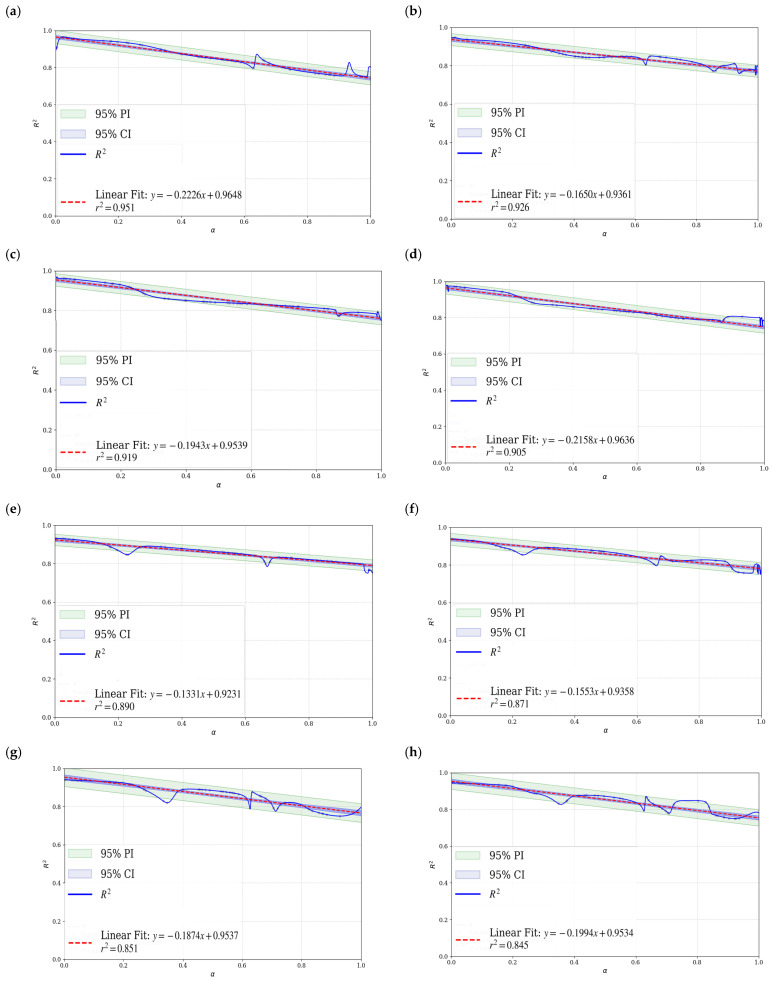
The variation patterns of the coefficient of determination (*R*^2^) values calculated based on FWO and Friedman methods under different reaction atmospheres with respect to the conversion rate: (**a**,**c**,**e**,**g**), respectively, represent the *R*^2^ changes of the FWO method for Air, 30% O_2_/70% CO_2_, 50% O_2_/50% CO_2_, and 70% O_2_/30% CO_2_, while (**b**,**d**,**f**,**h**), respectively, represent the *R*^2^ changes of the Friedman method for Air, 30% O_2_/70% CO_2_, 50% O_2_/50% CO_2_, and 70% O_2_/30% CO_2_. The blue solid line represents the smoothed longitudinal values of *R*^2^. The red dashed line represents the linear regression line of *R*^2^, with the fitting equation and the goodness-of-fit *r*^2^ labeled. The blue shaded area (95% CI) indicates the 95% confidence band. The green shaded area (95% PI) represents the 95% prediction band.

Figure 11 shows the variation patterns of the determination coefficient *R*^2^ values, the slope of the fitting line, and the statistical bandwidth with respect to α for different atmospheres of Air, 30% O_2_/70% CO_2_, 50% O_2_/50% CO_2_, and 70% O_2_/30% CO_2_ calculated by the FWO method (Figure 11a,c,e,g) and the Friedman method (Figure 11b,d,f,h), under different reaction atmospheres. The overall fitting correlation coefficients (*R*^2^) under each atmosphere remain at a relatively high level (usually exceeding 0.8) and fluctuate minimally, which fully demonstrates the excellent effect of linear fitting and the reliability of the calculation results. As the conversion rate α increases from 0 to 1, *R*^2^ shows a gradually decreasing trend overall; however, under high-oxygen-concentration conditions (such as 70% O_2_), this decrease is effectively controlled, ensuring the applicability and accuracy of the kinetic model throughout the reaction process. The red dotted line represents the linear regression line of *R*^2^ and indicates the fitting equation and the goodness of fit *r*^2^. As the oxygen concentration increases, the goodness of fit *r*^2^ of the regression line itself shows a slight decrease (for example, the FWO method decreases from 0.951 in air to 0.851 in 70% O_2_). This indicates that, in a high-oxygen environment, although the average *R*^2^ is still very high, the degree of dispersion of its distribution with respect to α has slightly increased. The narrow blue-shaded area (95% CI) closely adheres to the regression line, demonstrating a high positional certainty and minimal impact from random error. Under all atmospheres, the blue confidence band is extremely narrow and closely adheres to the fitted red line. This means that, regardless of changes in the reaction atmosphere, the large sample size and high fitting degree ensure that the certainty of the regression line position is extremely high, almost unaffected by random errors. The broad green-shaded area (95% PI) extensively encompasses the data points, reflecting the controlled data fluctuation and predictable deviation from the trend line. As the oxygen concentration increases, although the center position (regression line) of the prediction band is slightly adjusted, its width remains relatively stable, quantitatively demonstrating that the fluctuation range of the data relative to the fitted trend line is controlled and predictable under different atmospheres.

#### 3.4.2. Reaction Mechanism Analysis

**Figure 12:**
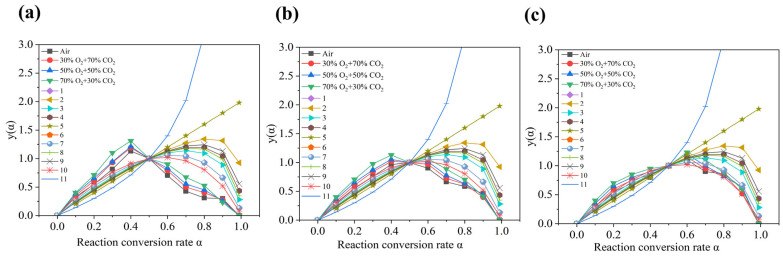
Curves of reaction mechanism functions under different heating rates and different atmospheres and their variations with reaction conversion rates: (**a**–**c**), respectively, represent the development trends of reaction mechanism functions at 10, 20, and 30 °C/min.

The determination of the mechanism function is based on the degree of similarity between the experimental curves obtained under air and various oxy-fuel combustion atmospheres and the theoretical standard curves of different kinetic models. The best-fitting standard curve, which exhibits the closest match to the experimental data, identifies the most probable reaction mechanism under the corresponding operating conditions, thereby elucidating the coupled interactions among mass transfer, heat transfer, and chemical reactions in the combustion process from a kinetic standpoint. At a pressure of 1 MPa, the mechanism functions of the RS combustion stage under three different heating rates were systematically investigated. As illustrated in Figure 12, the experimental curves are compared with standard theoretical curves across varying heating rates, in air, and in various oxy-fuel combustion atmospheres. Through an integrated analysis of the data in conjunction with Table 5, the most probable mechanism functions governing the RS combustion process under different atmospheric conditions were determined by evaluating both their integral and differential forms.

Based on the kinetic behavior of oxy-fuel combustion, the underlying mechanisms of mass diffusion and reaction interface evolution—such as diffusion control, phase boundary control, and nucleation and growth—were analyzed within the framework of gas–solid reaction models. As shown in Figure 12, the reaction conversion rate curve during RS combustion exhibits a distinct inflection point at a conversion degree α = 0.5, dividing the process into two characteristic stages: Combustion Stage I and Combustion Stage II. The mechanism function plots show consistent trends across different atmospheric conditions and predominantly align with theoretical curves 6, 7, and 10, which are typically associated with interfacial reaction mechanisms or diffusion-reaction coupled models. In the first combustion stage, increasing O_2_ concentration leads to a progressive deviation of the experimental curve from the theoretical profiles of curves 6, 7, and 10. In contrast, in the second combustion stage, higher O_2_ concentrations result in a gradual convergence of the experimental data toward these theoretical curves. With an increased heating rate, the experimental curve shows a closer alignment with the standard reference model. This behavior can be attributed to the combustion mechanism of RS, which involves the inward diffusion of O_2_ into the particle interior and the simultaneous outward diffusion of gaseous combustion products [63]. At lower heating rates (e.g., 10 °C/min), a significant temperature gradient develops between the sample’s interior and exterior. The resulting delay in heat transfer causes the asynchronous progression of the combustion reaction across different regions, leading to deviations from the uniform reaction process assumed by ideal chemical-reaction-controlled mechanism functions. However, when the heating rate is increased (e.g., 30 °C/min), the chemical reaction rate accelerates substantially, while diffusion remains relatively slower, though the associated lag time decreases. An enhanced external heat input enables the sample to reach the target temperature more rapidly, promoting a more uniform internal temperature distribution. Consequently, the reaction behavior approaches the idealized assumption of “global synchronization,” resulting in an improved agreement with the theoretical profile of the standard mechanism function.

Specifically, at heating rates of 10 °C/min and 20 °C/min, the experimental curves obtained under air and various oxy-fuel combustion atmospheres exhibit the closest agreement with curve 10. This correspondence indicates a reaction mechanism governed by three-dimensional diffusion, consistent with the Z-L-T equation. At a heating rate of 30 °C/min, under both air and an atmosphere composed of 30% O_2_ and 70% CO_2_, the experimental data exhibit the closest conformity to curve 10, consistent with the Z-L-T equation and indicative of a three-dimensional diffusion-controlled reaction mechanism. However, as the O_2_ concentration increases and the CO_2_ concentration decreases, particularly in atmospheres containing 50% O_2_/50% CO_2_ and 70% O_2_/30% CO_2_, the reaction behavior aligns more closely with the Avrami–Evans equation, suggesting a mechanism governed by random nucleation followed by growth, with the reaction order n ranging from 1/3 to 1/2 [64].

The combined effect of high heating rate and changes in oxygen concentration drives the transition from the “three-dimensional diffusion” mechanism to the “random nucleation” mechanism. The essence lies in the dynamic shift of the equilibrium point between the physical mass transfer resistance and the chemical reaction rate. At a low heating rate of 10–20 °C/min, due to the significant temperature gradient inside and outside the particles, the lag in heat transfer leads to an obvious asynchrony in the combustion reaction in different regions of the particles. At this time, it is difficult for the diffusion rate of oxygen towards the deeper part of the particles to match the consumption rate of the reaction, making physical diffusion the bottleneck that restricts the entire process, and, thus, presenting the typical three-dimensional diffusion mechanism (Z-L-T). In contrast, a high heating rate of 30 °C/min significantly shortens the time affected by mass transfer through a rapid external heat input, enabling the sample to rapidly reach the reaction temperature and tend towards uniformity. In this ideal reaction state approaching “global synchronization”, the interference of physical mass transfer resistance is effectively weakened, thereby providing the necessary physical prerequisite for the manifestation of the nucleation and growth mechanism.

This study demonstrates that the combustion reaction mechanism is not static but evolves dynamically in response to shifts in the balance between physical mass transfer resistance and chemical reaction kinetics. With an increasing oxygen concentration and heating rate, the governing kinetic regime transitions from three-dimensional diffusion-controlled behavior—described by the Z-L-T equation for intraparticle diffusion—to a mechanism dominated by random nucleation and subsequent growth, as characterized by the Avrami–Erofeev equation. At lower heating rates (10–20 °C/min), the oxygen diffusion coefficient in a CO_2_-rich atmosphere is approximately 70% of that observed in air. The pronounced thermal lag induces a significant asynchrony between the internal and external particle temperature fields, causing the oxygen penetration flux to substantially lag behind the oxygen consumption rate demanded by the surface reaction. Consequently, physical diffusion becomes the predominant rate-limiting step governing the overall reaction kinetics. In contrast, increasing the heating rate to 30 °C/min enhances the transient thermal input, thereby mitigating the cumulative effect of mass transfer resistance over time and promoting quasi-synchronous internal temperature distribution within the particles. Concurrently, the elevated oxygen partial pressure (50–70%) not only markedly increases the effective collision frequency of oxygen molecules at the reactive interface but also facilitates the in situ generation and rapid migration of reactive oxygen-centered radicals (e.g., O· and OH·). As a result, the apparent activation energy decreases by 47.5% relative to that under an air atmosphere. This shift drives the kinetic regime from diffusion-controlled to chemically controlled, with the rate-determining step transitioning from external oxygen mass transfer to nucleation and growth at catalytically active sites. Ultimately, the global reaction mechanism undergoes a robust transition.

**Figure 13:**
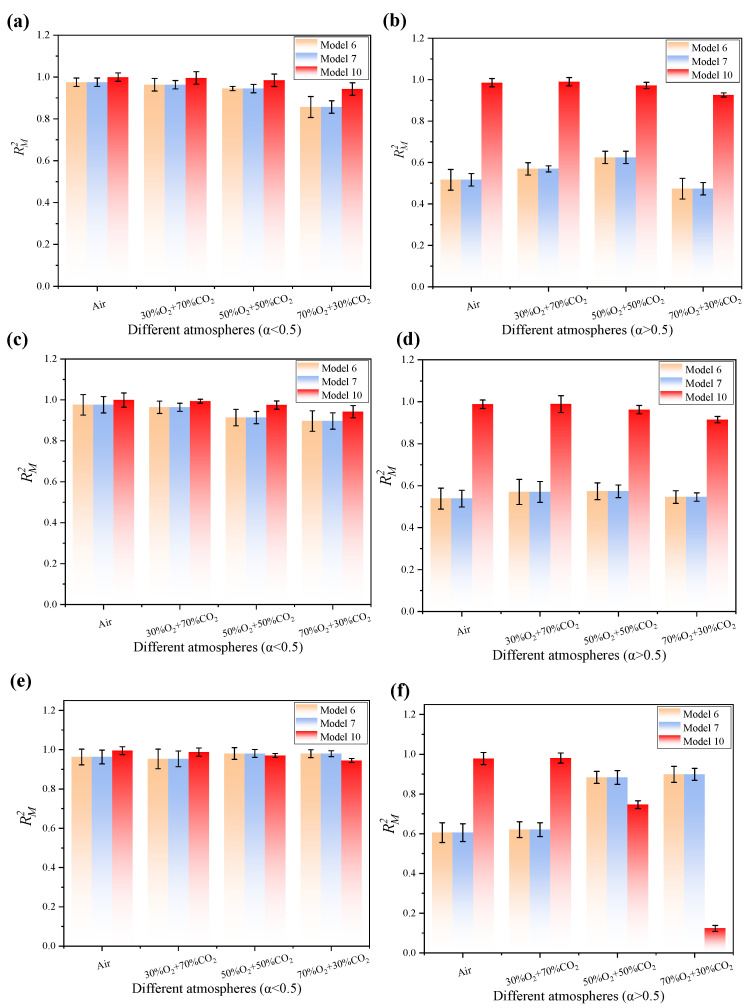
Curves of $R_{M}^{2}$ for different reaction mechanism functions under different heating rates with respect to the change of reaction conversion rate: (**a**,**c**,**e**), respectively, represent the development trend of $R_{M}^{2}$ for the reaction mechanism functions under the condition of α < 0.5 at heating rates of 10, 20, and 30 °C/min. (**b**,**d**,**f**), respectively, represent the development trend of $R_{M}^{2}$ for the reaction mechanism functions under the condition of α > 0.5 at heating rates of 10, 20, and 30 °C/min. The length of the error bars indicates the magnitude of the error.

Figure 13 shows the trend of $R_{M}^{2}$ for different models as the conversion rate changes. The statistical significance of $R_{M}^{2}$ is determined according to Formula (12), where $R_{M}^{2}$ measures the degree of overlap between the experimental observation curve and the theoretical prediction curve. The closer the value is to 1, the more accurately the kinetic model can describe the reaction process in the experiment. The stage-based mechanism analysis studies divide the combustion process into two stages with α = 0.5 as the boundary. The Figure 13a,c,e correspond to the first stage (α > 0.5), and the Figure 13b,d,f correspond to the second stage (α < 0.5).

In the first stage where α > 0.5, when the heating rate was increased to 30 °C/min, Figure 13 revealed a significant change in the kinetic mechanism. Although the reaction still favored curve 10 (three-dimensional diffusion) in air and 30%O_2_ atmospheres, at high oxygen concentrations of 50% and 70%, the random nucleation and subsequent growth model (such as the curve 6 or 7 corresponding to the Avrami–Erofeev equation) exhibited a higher $R_{M}^{2}$ goodness of fit.

In the second stage, where α > 0.5, the fitting results shown in Figure 13 indicate that, when the heating rate is at a relatively low level (10 °C/min and 20 °C/min), the Z-L-T equation (curve 10) has the highest $R_{M}^{2}$ value in the vast majority of atmospheres and is closest to 1. This data quantitatively proves that the reaction under these experimental conditions is mainly controlled by the three-dimensional diffusion mechanism, indicating that the diffusion and penetration process of oxygen into the particle interior constitutes the rate-limiting step of the entire combustion reaction.

Regarding the influence of the heating rate on the fitting quality, Figure 13 illustrates a crucial physical phenomenon: as the heating rate increases from 10 °C/min to 30 °C/min, the $R_{M}^{2}$ curve of the optimal fitting model becomes more stable and approaches 1. This indicates that, at higher heating rates, the enhanced external heat input promotes a more uniform internal temperature distribution within the sample, effectively eliminating the common internal thermal gradient interference observed during low-speed heating. Therefore, the statistical agreement between the experimental data and the ideal chemical reaction kinetics model has significantly improved. Figure 13 demonstrates that an increasing oxygen concentration leads to a marked reduction in the error bar width and a more homogeneous data distribution. These observations provide quantitative evidence of the enhanced measurement accuracy within this concentration range, thereby confirming the high reproducibility of the experimental system under these conditions.

## 4. Practical Application and Industrial Significance

This study establishes a rigorous cross-scale correlation framework grounded in the kinetic characteristics, ignition behavior, and complete combustion patterns observed in milligram-scale TG-DSC experiments. The framework serves as a quantitative criterion for interpreting micro-scale thermal decomposition and combustion dynamics in industrial thermal systems. By integrating volatile release kinetics with feedback-driven reaction modeling, the study bridges fundamental thermal data to practical engineering applications, specifically optimizing air distribution strategies in utility-scale boilers and guiding the design of heat transfer surfaces resistant to slagging. Consequently, it delivers essential parametric support for scaling up biomass carbon capture and storage (BECCS) technology from mechanistic understanding to megawatt-class demonstration. Addressing key operational challenges in oxy-fuel combustion, including thermal inertia and flame instability under high-CO_2_ atmospheres, the study uncovers a fundamental mechanistic consistency across spatial scales and validates the theoretical transition from the “three-dimensional diffusion-limited” regime to the “random nucleation and growth” regime. This insight enables an improved prediction of reaction rates and a robust steady-state control of large-scale power equipment under dynamic load conditions. Furthermore, through a systematic analysis of nitrogen- and sulfur-species conversion pathways, the study quantifies the energy-efficiency penalties inherent to BECCS-integrated biomass energy systems and strengthens the scientific basis for deploying biomass oxy-fuel combustion at scale, thereby advancing the power sector’s pathway toward net-negative carbon emissions.

Although this study deeply revealed the essential kinetic characteristics of RS in an oxygen-rich environment through thermogravimetric analysis and differential scanning calorimetry and differential scanning calorimetry (TG-DSC), there are still certain limitations that need to be further improved in subsequent work. Firstly, the laboratory-scale TG device operates under constant pressure, which cannot fully simulate the complex pressure fluctuations and intense turbulence effects in industrial boilers. This leads to deviations in the gas–solid mass transfer rate from the actual working conditions. Secondly, although the specific crucible diffusion limitation of the thermal gravimetric system has been minimized as much as possible in the calculation, there are still essential differences between its static environment and the complex flow fields in fluidized beds and other dynamic reactors. Therefore, the kinetic models and pollutant transformation laws obtained in this study mainly serve as basic theoretical references. Future research will focus on verifying them on pilot-scale platforms, introducing multi-phase flow coupling and pressure gradient parameters, in order to achieve a precise transition from laboratory basic research to complex industrial application scenarios.

## 5. Conclusions

This study systematically explored the influence of an oxygen-rich atmosphere on the combustion behavior of RS. By leveraging TG, DSC, and MS analysis, this research offers robust empirical evidence for an improved combustion performance and emission control. It was observed that oxy-fuel combustion, especially at high oxygen levels, markedly reduced fuel ignition and burnout temperatures compared to air. The study explicitly defined the optimal combustion environment for RS as 70% O_2_/30% CO_2_ at a heating rate of 30 °C/min. In this optimized state, the fuel exhibited a sixfold enhancement in (dw/dt)_max_ over air conditions and a substantial 152 °C decrease in burnout temperature T_b_, confirming the highly intensified nature of the reaction. Moreover, due to the higher density and heat capacity of CO_2_, which enhanced the thermal inertia of the system, the combustion process exhibited stronger stability. The mass spectrometry analysis further confirmed that the combination of the oxygen-rich environment and a high heating rate inhibits the formation of thermal NO and collaboratively suppresses the release of sulfur oxides. The reduction in NO_2_ was over 90%, NO was reduced by approximately 75%, SO_2_ was reduced by 65%, and COS was reduced by 80%, achieving a simultaneous improvement in combustion intensity and environmental performance.

At the level of kinetic analysis, this study utilized FWO and Friedman’s universal methods to explain the profound transformation of the reaction mechanism in response to the environmental atmosphere from a mathematical model perspective. The results indicated that oxy-fuel combustion significantly lowered the apparent activation energy E compared to air. Specifically, the average E value determined by the FWO method dropped sharply from 210.5 kJ/mol to around 110.5 kJ/mol, representing a 47.5% decrease. Such a dramatic decline effectively lowers the energy threshold for the combustion reaction, demonstrating the superior catalytic or promotional effects of high oxygen concentrations on biomass thermal degradation. The study importantly discovered that the reaction mechanism is not constant but is driven by the balance between the physical mass transfer and chemical reaction rate: at low oxygen concentrations or slow heating rates, due to the limitation of oxygen diffusion into the particles, the reaction follows the Z-L-T three-dimensional diffusion model, while, in the coupling of high oxygen and rapid heating, the sufficient oxygen supply enables the chemical reaction to break through the mass transfer bottleneck, and the mechanism shifts to follow the Avrami–Erofeev equation’s stochastic nucleation and subsequent growth model.

The oxy-fuel combustion of RS not only achieves “near-zero emissions”, but is also the core for achieving “negative emissions” in carbon neutrality. The established kinetic model by the research institute can directly guide the air distribution design, heat transfer surface layout, and the risk control of alkali metal ash accumulation in industrial boilers, providing feasible engineering guidance and clean utilization solutions for the low-carbon transformation in the power and industrial sectors. It is crucial to acknowledge the inherent disparities in heat transfer dynamics between the micro-gram scale of TG-DSC measurements and the complex, large-scale environment of industrial boilers. Future research should focus on the evolution of ash accumulation and slag formation caused by AAEMs in pilot-scale fluidized beds to enhance engineering applicability.

## Figures and Tables

**Table 1:** Ultimate analysis, atomic ratio of RS.

Samples	Ultimate Analysis					H/C	O/C	(O + N)/C
	C(%)	H(%)	N(%)	S(%)	O(%) ^a^			
RS	38.67 ± 1.98	4.94 ± 0.11	1.89 ± 0.01	0.67 ± 0.07	42.42 ± 2.19	1.53 ± 0.11	0.82 ± 0.17	0.86 ± 0.04

^a^ Calculated by difference: O(%) = 100% − C(%) − H(%) − N(%) − S(%) − A(%) − M(%).

**Table 2:** Proximate analysis, fuel ratio, lignocellulosic components, and HHV of RS.

Samples	Proximate Analysis				Fuel Rate ^b^	Lignocellulosic Components (wt. %)			HHV (MJ/kg)
	VM(%)	A(%)	FC(%)	M(%)		Cellulose	Hemicellulose	Lignin	
RS	71.03 ± 2.13	5.05 ± 0.11	17.56 ± 0.29	6.36 ± 0.21	0.25 ± 0.01	38.32 ± 0.53	25.11 ± 0.29	12.55 ± 0.61	14.59 ± 0.45

^b^ Fuel rate = FC/VM.

**Table 3:** Ash content determination of RS.

Composition	SiO_2_	Al_2_O_3_	GaO	Fe_2_O_3_	SO_3_	K_2_O	Na_2_O	TiO_2_	MgO	P_2_O_5_
Content	46.62	3.8	7.78	3.83	1.06	19.41	1.92	0.19	7.67	2.15

**Table 4:** Combustion characteristic parameters of RS under different heating rates and different atmospheres.

Combustion Conditions Parameters		*T_i_* (°C)	*T_b_* (°C)	*T_f_* (°C)	*t_i_* (Min)	*t_b_* (Min)	Δ*t*_1/2_ (Min)	(*dw*/*dt*)*_max_* (%/Min)	(*dw*/*dt*)*_mea_*_n_ (%/Min)	*t_max_* (Min)	*C_i_* (%/Min ^3^)	*C_b_* (%/Min ^4^)	*S* (%/°C ^3^ × Min ^2^)
10 °C/min	Air	287.1	467.5	320.1	20.2	38.3	20.9	−11.3	4.3	23.5	−0.02380	−0.00070	1.26095 × 10^−6^
	30% O_2_ + 70% CO_2_	271.8	369.5	310.2	18.6	28.5	22.1	−28.5	8.5	22.5	−0.06810	−0.00243	8.87463 × 10^−6^
	50% O_2_ + 50% CO_2_	266.5	346.2	300.1	18.1	26.1	21.4	−49.5	10.6	21.5	−0.12720	−0.00490	2.13398 × 10^−5^
	70% O_2_ + 30% CO_2_	260.4	332.1	295.5	17.5	24.7	20.8	−65.6	11.9	21.0	−0.17850	−0.00730	3.46657 × 10^−5^
20 °C/min	Air	305.3	492.5	340.5	11.3	20.4	11.5	−23.6	8.9	12.8	−0.16316	−0.00890	4.57554 × 10^−6^
	30% O_2_ + 70% CO_2_	290.6	429.4	330.7	10.3	17.2	12.2	−112.2	12.1	12.3	−0.88563	−0.05191	3.74391 × 10^−5^
	50% O_2_ + 50% CO_2_	283.2	349.5	320.3	9.9	13.2	11.7	−137.6	25.2	11.8	−1.17788	−0.09000	1.23704 × 10^−4^
	70% O_2_ + 30% CO_2_	279.1	340.5	315.0	9.7	12.8	11.6	−144.2	27.6	11.5	−1.29269	−0.10012	1.50051 × 10^−4^
30 °C/min	Air	323.6	506.5	360.1	7.9	14.1	8.5	−38.7	12.4	9.2	−0.53247	−0.04087	9.04766 × 10^−6^
	30% O_2_ + 70% CO_2_	309.7	490.1	350.2	7.5	13.5	8.8	−285.9	13.4	8.8	−4.33182	−0.32088	8.14989 × 10^−5^
	50% O_2_ + 50% CO_2_	301.3	477.2	340.1	7.2	13.1	8.5	−342.6	13.9	8.5	−5.59804	−0.42733	1.10993 × 10^−4^
	70% O_2_ + 30% CO_2_	295.2	466.5	335.1	7.5	12.7	8.3	−432.3	14.4	8.3	−6.94458	−0.54682	1.53131 × 10^−4^

**Table 5:** Commonly used kinetic mechanism functions under different heating rates and different atmospheres.

Number	Function Name	Mechanism	Integral Form *G*(*α*)	Differential Form *f*(*α*)
1	Mample, First Grade	Random nucleation and subsequent growth	−ln(1 − *α*)	1−*α*
2	Valensi Equation	Two-dimensional diffusion with cylindrical symmetry	*α +* (1 − *α*)ln(1 − *α*)	[−ln(1 − *α*)]^−1^
3	Contraction sphere	Two-phase boundary reaction, spherically symmetric, *n* = 1/3	1 − (1 − *α*)^1/3^	3(1 − *α*)^2/3^
4	Contraction cylindrical shape	Phase boundary reaction, cylindrical symmetry, *n* = 1/2	1 − (1 − *α*)^1/2^	2(1 − *α*)^1/2^
5	Parabolic rule	One-dimensional diffusion	*α* ^2^	1/2*α*^−1^
6	Avrami–Evofee Equation	Random nucleation followed by growth, *n* = 1/2	[−ln(1 − *α*)]^1/2^	2(1 − *α*)[−ln(1 − *α*)]^1/2^
7	Avrami–Evofee Equation	Random nucleation followed by growth, *n* = 1/3	[−ln(1 − *α*)]^1/3^	3(1 − *α*)[−ln(1 − *α*)]^2/3^
8	Jander Equation	Three-dimensional diffusion, spherical symmetry, 3D	[1 − (1 − *α*)^1/3^]^2^	3/2(1 − *α*)^2/3^[1 − (1 − *α*)^1/2^]^−1^
9	G-B Equation	Three-dimensional diffusion, cylindrical symmetry, 3D	1 − 2*α*/3 − (1 − *α*)^2/3^	3/2[(1 − *α*)^−1/3^ − 1]^−1^
10	Z-L-T Equation	Three-dimensional diffusion, 3D	[1 − (1 − *α*)^−1/3^]^2^	3/2(1 − *α*)^4/3^[(1 − *α*)^−1/3^ − 1]^−1^
11	Reaction order	*n* = 2	1 − (1 − *α*)^2^	1/2(1 − *α*)^−1^

## Data Availability

The original contributions presented in the study are included in the article. Further inquiries can be directed to the corresponding author.
